# Exploring Neuroprotective Potential of Bioactive Compounds Obtained from Artichoke By-Products by Pressurized Liquid Extraction via Response Surface Methodology

**DOI:** 10.3390/ijms27094059

**Published:** 2026-04-30

**Authors:** Edmondo Messinese, Alberto Valdés, Antonella Cavazza, Alejandro Cifuentes

**Affiliations:** 1 Dipartimento di Scienze Chimiche, Della Vita e della Sostenibilità Ambientale (SCVSA), Università di Parma, Parco Area delle Scienze 17/A, 43124 Parma, Italy; edmondo.messinese@unipr.it (E.M.); antonella.cavazza@unipr.it (A.C.); 2 Foodomics Laboratory, Institute of Food Science Research (CIAL), CSIC-UAM, Nicolas Cabrera 9, 28049 Madrid, Spain; a.cifuentes@csic.es

**Keywords:** agro-food by-products, blood–brain barrier, caffeoylquinic acids, green extraction, neuroprotective activity, pressurized liquid extraction

## Abstract

Artichoke by-products (ABP) represent valuable sources of bioactive compounds with relevant health benefits. In this study, a green extraction strategy based on pressurized liquid extraction (PLE) was optimized to enhance the recovery of phenolic and flavonoid compounds from ABP using a response surface methodology. Extraction temperature and solvent composition were identified as the key factors driving extraction performance. Optimal conditions using a mixture of ethyl acetate and ethanol (90/10, *v*/*v*) at 180 °C significantly enhanced extraction yield, total phenolic and flavonoid content, and antioxidant activities, as measured by ORAC and DPPH assays. Chemical characterization via HPLC-C18-Q-TOF-MS/MS revealed a diverse profile of phenolic and flavonoid compounds, including caffeoylquinic acid derivatives and related transformation products. The neuroprotective potential of the optimized extract was further evaluated through in vitro inhibition assays targeting acetylcholinesterase (AChE), butyrylcholinesterase (BChE) and lipoxygenase (LOX), alongside a permeability assessment using an in vitro blood–brain barrier (BBB) model. Molecular docking simulations were performed to explore the interactions of apigenin—the most representative flavonoid in the optimal extract—with the three target enzymes. Overall, these findings support the valorization of ABP as a source of bioactive compounds and highlight the potential of PLE as an efficient and sustainable extraction approach.

## 1. Introduction

Artichoke (*Cynara scolymus* L.) is a perennial herbaceous plant belonging to the Asteraceae family, widely cultivated in the Mediterranean region [1]. Traditionally consumed as a culinary delicacy, it is valued for its high nutritional content and health-promoting properties. Its therapeutic properties have been known since ancient Egyptian, Greek, and Roman times, when it was used as a medicine to treat liver and digestive disorders [2]. According to FAOSTAT data [3], the Mediterranean region produced approximately 1.5 million metric tons of artichokes in 2023, representing 80% of global production. The European Union accounts for 38.5% of total world production, with Italy, Spain and France being the main producers [3]. Artichokes have been extensively characterized due to their richness in bioactive compounds such as phenolics, flavonoids, inulin, and pectin [1]. These bioactive molecules confer a wide range of biological activities to artichokes, including antioxidant [4,5], anti-inflammatory [6,7,8], anti-microbial [8,9], hepatoprotective [4,6], cardioprotective [10,11], and neuroprotective effects [6,8,11,12,13,14], making them valuable for various applications in the food, pharmaceutical, and cosmetic sectors [15,16,17,18].

Artichoke by-products (ABP), primarily composed of external bracts, represent approximately 60–80% of the total biomass generated during industrial processing [1]. These by-products, rich in bioactive compounds such as phenolics and flavonoids, are underutilized and pose significant environmental and economic challenges [19]. This is particularly unfortunate as they possess significant biotechnological and nutritional potential which can be effectively exploited within a circular economic framework [19]. Among the various by-products, external bracts are the primary component, which is noteworthy as they are still rich in bioactive molecules [18]. Given the vast quantities of these agro-food by-products, they represent an underexploited source of bioactive compounds with significant biotechnological and nutritional potential that can be effectively exploited within a circular economy framework [20].

ABP are recognized for their high content of phenolic and flavonoid compounds, with hydroxycinnamic acids derived from caffeic acid being the predominant phenolic constituents. These compounds are primarily present in conjugation with quinic acid, forming caffeoylquinic derivatives, among which cynarin (1,3-O-dicaffeoylquinic acid), chlorogenic acid (3-O-caffeoylquinic acid), neochlorogenic acid (5-O-caffeoylquinic acid), and 1,5-O-dicaffeoylquinic acid are the most notable [1]. Flavonoids represent the second most abundant class, with flavones such as apigenin, luteolin, and their glycoside and rutinoside derivatives being particularly prominent [1].

The high content of bioactive molecules in artichoke extracts confers significant antioxidant potential, promoting the scavenging of reactive oxygen species (ROS) and reactive nitrogen species (RNS), thereby mitigating oxidative damage in biological systems [1,21]. Administration of artichoke extracts was found to significantly reduce oxidative stress markers, particularly in H_2_O_2_-induced HepG2 cells [22,23]. Notably, artichoke extract treatment reduced intracellular ROS levels and enhanced antioxidant enzyme activities, including superoxide dismutase and catalase, thereby promoting cellular resilience to oxidative insults [20,24]. Bioactive compounds found in artichoke have also been reported to exert high neuroprotective potential [1]. A critical aspect of this potential is their inhibitory activity against acetylcholinesterase (AChE) and butyrylcholinesterase (BChE), enzymes involved in the degradation of acetylcholine. Dysregulation of these enzymes is associated with neurodegenerative disorders such as Alzheimer’s and Parkinson’s diseases, as well as psychiatric conditions including schizophrenia and depression [25,26]. Thus, dual inhibition of both enzymes offers a strategic therapeutic approach to mitigate the cognitive deficits associated with these disorders. Flavonoids such as quercetin, apigenin, and luteolin have been described to ameliorate cognitive deficits by modulating oxidative stress and inflammatory pathways [1,12]. In addition, artichoke extracts have demonstrated pronounced anti-inflammatory effects, mainly mediated by inhibition of key enzymes involved in inflammatory pathways, such as cyclooxygenase (COX) and lipoxygenase (LOX), which are responsible for the production of pro-inflammatory mediators, including prostaglandins and leukotrienes associated with chronic inflammation and oxidative stress [5].

Conventional extraction methods have been widely used for decades to isolate bioactive molecules from plant matrices. However, these traditional approaches often involve long extraction times, high solvent consumption, and considerable energy demands, posing both economic and environmental challenges [27]. Novel extraction techniques have gained considerable attention due to their enhanced efficiency and reduced environmental impact. These methods are designed to optimize the recovery of bioactive compounds from agro-food by-products, leveraging advanced technologies such as supercritical fluid extraction (SFE) [27,28], ultrasound-assisted extraction (UAE) [29,30,31], and pressurized liquid extraction (PLE) [14,29,32]. Among these, PLE takes advantage of high pressures and moderate temperatures to improve solvent penetration and mass transfer, making it particularly suitable for the recovery of bioactive compounds such as polyphenols, flavonoids, carotenoids, and other valuable molecules from plant residues [32]. The integration of PLE with analytical techniques, such as high-performance liquid chromatography (HPLC) and mass spectrometry (MS), has been reported to enhance the identification and quantification of extracted compounds, thereby supporting the development of high-quality natural products [33,34,35]. In this study, the optimal PLE conditions to obtain bioactive compounds from ABP, consisting of external bracts, were determined using response surface methodology (RSM) based on a central composite design (CCD), optimizing both extraction temperature and solvent composition. The extracts obtained were extensively analyzed by in vitro assays to evaluate key parameters describing bioactive compound contents and antioxidant activities. Specifically, total phenolic content (TPC), total flavonoid content (TFC), oxygen radical absorbance capacity (ORAC), and 2,2-diphenyl-1-picrylhydrazyl (DPPH) radical scavenging activity were considered. A chemical characterization analysis using UHPLC-Q-TOF-MS/MS was adopted to investigate the phenolic and flavonoid profiles present in ABP PLE extracts obtained under optimized conditions. In vitro neuroprotective potential was evaluated by AChE and BChE inhibitory assays and anti-inflammatory assays via LOX inhibition. Moreover, a parallel artificial membrane permeability assay (PAMPA) was performed to assess the permeability of the bioactive compounds across an artificial blood–brain barrier (BBB). Finally, molecular docking simulations were conducted to explore the interactions between the active sites of AChE, BChE, and LOX enzymes with apigenin, the most interesting bioactive compound identified in the optimal ABP PLE extract. To the best of our knowledge, this is also the first study reporting the formation of ethyl caffeoylquinic acid esters and caffeoylquinic acid lactones in artichoke by-products under PLE conditions.

## 2. Results and Discussion

### 2.1. Green Solvents Selection for PLE

**Figure 1:**
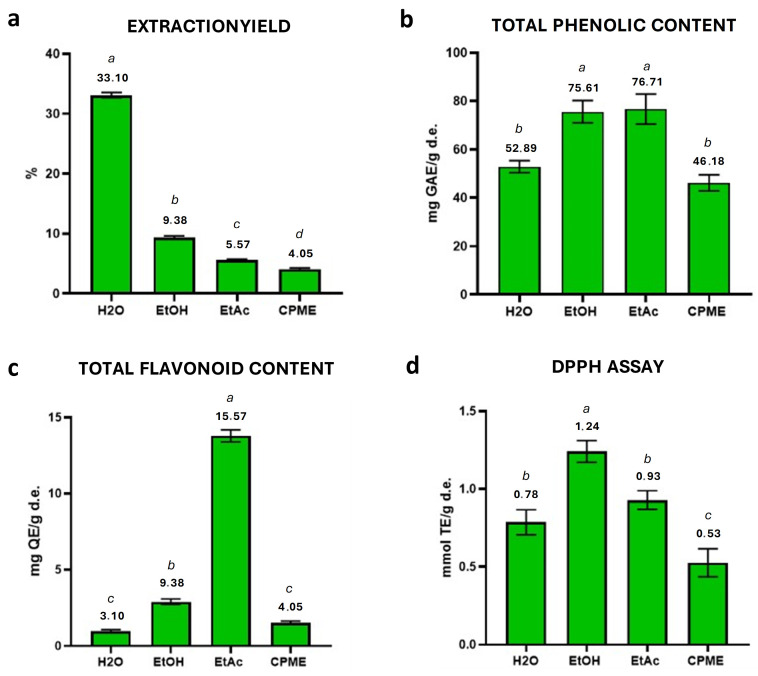
(**a**) Extraction yield, (**b**) total phenolic content, (**c**) total flavonoid content and (**d**) DPPH results of PLE extracts obtained using H_2_O, EtOH, EtAc and CPME from artichoke by-products using 10.34 MPa, 115 °C and 1 cycle of extraction for 20 min. Different letters (a, b, c, d) above bars indicate statistically significant differences between solvents within the same response variable (one-way ANOVA followed by Tukey’s post hoc test, *p* < 0.05). “d.e.” denotes dry extract.

The extraction of bioactive compounds from ABP was initially performed by PLE using various green solvents with different polarities (water (H_2_O), ethanol (EtOH), ethyl acetate (EtAc) and cyclopentyl methyl ether (CPME)), all of which are Generally Recognized as Safe (GRAS) and suitable for food purposes [21]. To this end, their extraction efficacy in maximizing extraction yield, TPC, TFC, and minimizing DPPH values (indicating better antioxidant activity expressed as IC_50_) was evaluated and the results are shown in Figure 1.

Water yielded the highest extraction efficiency, likely due to its ability to dissolve a broad range of chemical compounds from the matrix, including phenolic acids, flavonoids, and hydroxycinnamic acid derivatives. However, the TPC, TFC and DPPH values obtained from pressurized water extraction were significantly lower than those obtained from the other solvents. This behavior can be attributed to the different degree of polarity, which improves the solubilization of phenolic compounds [36]. Notably, extraction yield reflects the total amount of soluble solids, including non-phenolic constituents (e.g., sugars and proteins), and therefore does not necessarily correlate with TPC, TFC, or antioxidant activity [37].

In contrast, EtOH and EtAc provided superior performance in terms of phenolic and flavonoid content (Figure 1b,c). On the other hand, CPME was evaluated for its ability to extract non-polar compounds but showed lower extraction performances. Based on these results, EtOH and EtAc solvents were selected for further optimization of the PLE method [38,39].

### 2.2. PLE Optimization by Response Surface Methodology (RSM)

Based on the results obtained with EtOH and EtAc, PLE conditions were optimized using a response surface methodology (RSM) based on a Central Composite Design (CCD) to enhance the extraction of bioactive compounds from ABP. The experimental design utilized a three-level factorial approach, examining two independent variables: solvent composition (0%, 50%, and 100% of EtOH in EtAc) and temperature (50, 115, and 180 °C). For antioxidant assays, IC_50_ values were used as inverse responses, as lower values indicate higher antioxidant capacity, and were incorporated into the desirability function accordingly. This design enabled the evaluation of the effects on extraction performance, aiming to maximize response variables such as extraction yield, TPC, and TFC, while minimizing other response variables associated with antioxidant capacity, such as DPPH and ORAC (where a better antioxidant capacity is indicated by a lower IC_50_).

As shown in Table 1, higher extraction temperatures resulted in better recovery values (runs 1, 7, 8 and 11), whereas lower extraction yields were observed at lower temperatures. Moreover, extraction yield was also enhanced as EtOH increased (runs 8, 9 and 11). These responses were significantly different based on the RSM (Table S1, Supplementary Material). Regarding phenolic and flavonoid content, TPC was mainly driven by extraction temperature (Table S2, Supplementary Material), whereas TFC was predominantly influenced by solvent composition (Table S3, Supplementary Material). Notably, increasing the EtAc percentage resulted in the highest TPC and TFC values (run 1), indicating the strong chemical affinity of these bioactive compounds for EtAc. These findings are consistent with previous studies [40,41], confirming that the combination of EtAc and elevated temperatures is particularly effective for extracting slightly polar and non-polar compounds, such as phenolics and flavonoids, which are associated with high antioxidant potential. The influence of temperature and solvent composition was also evident in the IC_50_ values related to ORAC and DPPH assays. Higher extraction temperatures were linked to lower IC_50_ values, with optimal results achieved at 180 °C. In the ORAC assay, the highest values were observed when increasing the EtAc percentage, resulting in an IC_50_ of 1.5 μg/mL. Similarly, the DPPH assay showed optimal radical scavenging activity at 180 °C with an increasing percentage of EtAc (run 1 and 7). Antioxidant responses showed a strong dependence on both temperature and solvent composition, including significant quadratic and interaction terms (Tables S4 and S5, Supplementary Material). Pareto charts further highlighted temperature as the dominant factor for most response variables, except for TFC, where solvent composition played the primary role, in agreement with ANOVA outcomes (Figure S1, Supplementary Material).

Theoretical predictions for optimal PLE conditions were calculated based on the obtained results (see Table 1), integrating all response variables through a desirability function (Figure S2, Supplementary Material). The predictions indicated optimal extraction conditions at a temperature of 180 °C using an EtAc-rich solvent mixture (EtAc:EtOH, 90:10, *v*/*v*), resulting in a desirability score of 84.4%. These theoretical predictions were experimentally validated (*p* > 0.05) by performing extractions under the proposed conditions and assessing the same response variables (Table 2). The experimental results were consistent with the predicted values, confirming the accuracy of the regression models in forecasting response outcomes.

Although these conditions maximize phenolic recovery, high temperatures may affect compound stability. While they enhance mass transfer [32], they can also promote the degradation of thermolabile polyphenols and the generation of thermally induced transformation products, such as caffeoylquinic acid lactones and ethyl ester adducts in the optimized ABP extract (see Section 2.4). These reactions, previously reported under similar conditions [42,43], may alter extract composition and bioactivity. Therefore, strict temperature control is essential to ensure reproducibility and consistent product profiles. These observations imply that the bioavailability and biological activity of the bioactive compounds occurring in the extract may differ from that of conventionally extracted material, since the native compound profile is partially altered. This underlines the importance of instrument calibration and robust method standardization for any scale-up or inter-laboratory application of the protocol.

The PLE-optimized extract was also assessed for its pigment contents through spectrophotometric assays. Specifically, data revealed a total chlorophyll content of 15.25 + 0.67 μg/g and a total carotenoid content of 1.62 ± 0.16 μg/g, both markedly higher than previous values reported by using UAE. According to a previous work [31], the highest total chlorophyll and carotenoid contents achieved via UAE were 10.68 μg/mL and 0.57 μg/mL, respectively, with a reduction in yields at longer extraction times. This contrast highlights the superior efficiency of PLE in extracting pigments, likely due to the use of elevated temperature and pressure, which can enhance solvent performance [32]. Additionally, the optimized PLE conditions yielded a significantly higher TPC (165.82 ± 1.24 mg GAE/g) compared to UAE, PLE and maceration techniques [31,40,41,44], further demonstrating its effectiveness in recovering bioactive compounds.

Complementary experiments were also conducted using ternary solvent mixtures (EtAc/EtOH/H_2_O at 33:33:33 *v*/*v*/*v* and EtAc/EtOH/CPME at 33:33:33 *v*/*v*/*v*) at 180 °C. The inclusion of H_2_O led to a statistically significant decrease in TPC (142.4 ± 1.2 mg GAE/g d.e.), antioxidant capacity (ORAC IC_50_: 10.4 ± 0.5 μg/mL), and cholinesterase inhibitory activity (AChE IC_50_: 596.4 ± 8.1 μg/mL), suggesting that water promotes thermo-oxidative degradation of sensitive bioactive compounds under PLE conditions [38,39] (see Table S6, Supplementary Material). Similarly, total chlorophyll content was substantially reduced in the ternary water-containing mixture (12.6 ± 0.7 μg/g d.e.), further supporting the deleterious effect of aqueous phases at elevated temperatures (see Table S7, Supplementary Material).

### 2.3. In Vitro Neuroprotective Potential Evaluation

**Figure 2:**
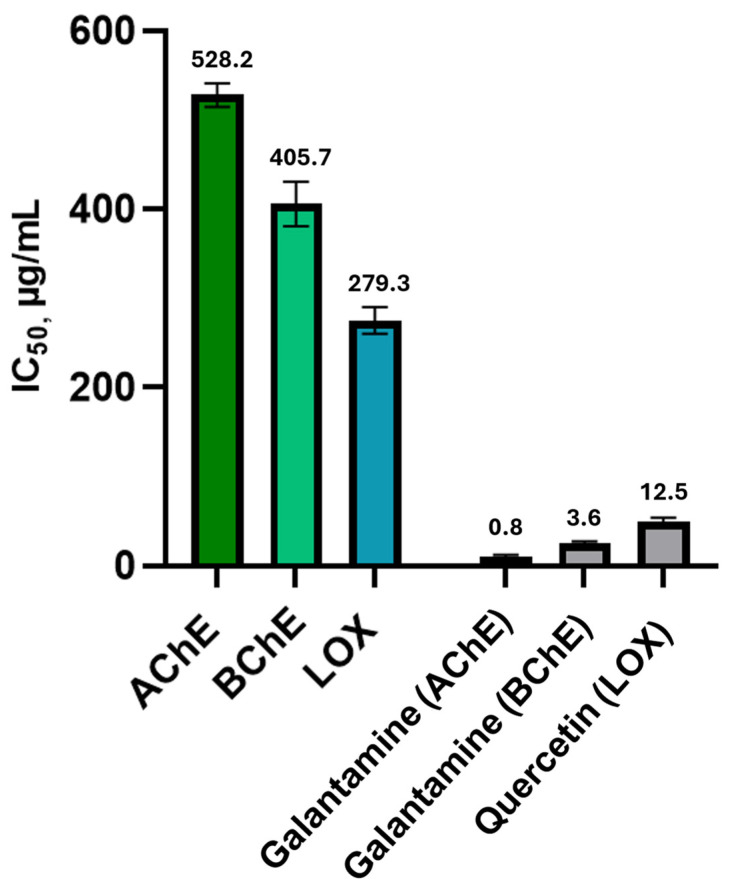
Neuroprotective potential evaluation by inhibition assays of AChE, BChE, and LOX for artichoke by-products (ABP) PLE optimal extract. Galantamine was used as a positive control for AChE (IC_50_ = 0.8 ± 0.2) and BChE (IC_50_ = 3.6 ± 0.2) inhibition; quercetin was used as a positive control for LOX inhibition (IC_50_ = 12.5 ± 0.5).

The neuroprotective potential of the ABP extract obtained under optimized PLE conditions was evaluated through in vitro enzyme inhibition assays targeting AChE, BChE, and LOX. These enzymes are critically implicated in neurodegenerative and inflammatory processes associated with disorders such as Alzheimer’s disease, making them valuable biological targets. The results were expressed as IC_50_ values (concentration of extract providing 50% inhibition): lower IC_50_ values relate to a stronger neuroprotective and anti-inflammatory efficacy. The results of anti-cholinergic and anti-lipoxygenase activities are shown in Figure 2.

The ABP extract showed moderate AChE and BChE inhibition (IC_50_ = 523 and 405 μg/mL), markedly weaker than galantamine, and lower values than those reported for fresh artichoke extracts (e.g., Spinoso Sardo and Romanesco Siciliano) [8]. This reduced potency likely reflects matrix dilution effects and the use of industrial by-products, which may contain lower levels of active flavonoids. Processing-related degradation altered phytochemical synergy and accumulation of inactive components may contribute further [45]. Despite these effects, the ABP extract retains measurable bioactivity, supporting the valorization of ABP within a green extraction framework. HPLC-C18-Q-TOF MS/MS analysis (Section 2.4) identified compounds such as apigenin and luteolin, known cholinesterase inhibitors, whose activity is linked to structural features (e.g., catechol groups, conjugated systems) that enable interaction with enzyme active sites [46,47].

The anti-inflammatory activity of the ABP extract was assessed by LOX inhibition, revealing an IC_50_ value of 275 μg/mL. Although moderate, this activity is consistent with the antioxidant profile of the extract and supports its potential to mitigate neuroinflammation, a major contributor to neurodegenerative progression. Flavonoids such as apigenin-7-glucoside, previously identified in artichoke leaves [48], are known to specifically inhibit LOX and COX pathways, reinforcing the relevance of artichoke-derived metabolites in controlling inflammatory cascades. Nonetheless, the slightly reduced neuroprotective potency observed in ABP compared to primary agricultural products could be related to processing-induced degradation of sensitive compounds, alteration of synergistic interactions among phytochemicals, and the accumulation of non-beneficial components during industrial processing [45]. These findings highlight the necessity for optimized post-harvest and storage strategies to preserve the functional integrity of bioactive compounds in agro-industrial residues.

Overall, ABP extracts represent a viable source of neuroactive compounds, and PLE provides an efficient and sustainable recovery strategy. However, safety remains a critical consideration. While major constituents (caffeoylquinic acids, apigenin, luteolin and derivatives) are well-established dietary compounds with favorable safety profiles [1,2,18], the optimized PLE conditions (180 °C, EtOH/EtAc 10:90 *v*/*v*) promote the formation of non-native products, including CQA lactones and ethyl esters (Section 2.4), whose toxicological profiles are unknown. High-temperature extraction may also concentrate minor undesired compounds (e.g., oxidation or Maillard-derived products). Accordingly, further work should include in vitro cytotoxicity assays (e.g., MTT) and in vivo safety studies to support future applications.

### 2.4. Chemical Characterization of ABP PLE Optimal Extract by HPLC-C18-Q-TOF MS/MS

Chemical characterization analysis by HPLC-Q-TOF MS/MS was carried out to investigate the phenolic and flavonoid profiles of ABP extracts obtained from PLE under optimal conditions. The analysis was performed in ESI (+) and ESI (−) ionization modes, leading to the tentative identification of 39 compounds. The assignment of each metabolite was possible by comparing the exact mass (*m*/*z*), the retention times and MS/MS spectra with standards, existing databases and references from the literature. Table 3 presents the tentative identifications of metabolites in ABP, including their retention times, experimental *m*/*z*, proposed formulas, corresponding chemical families, and main MS/MS fragments.

As it can be observed, the identity of seven compounds was confirmed by using standards (protocatechuic acid, chlorogenic acid, luteolin-7-O-glucoside, apigenin 7-O-glucoside, ethyl caffeate, luteolin and apigenin), and up to 32 compounds belonging to different families were tentatively identified. As expected, most of these compounds were detected using ESI (−) ionization mode. However, two flavonoids (luteolin 7,3′-dimethyl ether and 4′,7-dimethoxy-3-hydroxyflavone) were detected as [M+H]^+^ ions due to the presence of methoxy (–OCH_3_) groups, whose fragmentation can be more informative than that generated from the [M-H]^−^ ions [49,50]. The predominant category was that of phenolic compound derivatives (*n* = 27), followed by flavonoid derivatives (*n* = 8). The different phenolic compounds were mainly attributed to benzoic and hydroxycinnamic acids (such as caffeic acid, mono- and di-caffeoylquinic acids and their derivatives, and ethyl caffeate), while the main flavonoids were apigenin, luteolin, luteolin 7,3′-dimethyl ether, and their corresponding glucoside derivatives. Many of these compounds have already been described in the scientific literature and are well-known to contribute to TPC and TFC, as well as to possess antioxidant capacity [1]. Most of these compounds have also demonstrated high potential as drug candidates for neurodegenerative disease treatments, as they have been reported to promote neuroprotective [8,14,15,51] and anti-inflammatory effects [8,15,51,52].

**Table 3:** Tentatively identified compounds in ABP PLE extracts obtained by HPLC-C18-Q-TOF MS/MS ESI (+/−) analyses. Row shading indicates chemical family: light blue = benzoic acids and hydroxyphenols; light yellow = hydroxycinnamic acids (mono-caffeoylquinic acid derivatives); light orange = hydroxycinnamic acids (di-caffeoylquinic acid derivatives); light green = flavonoids; light purple = lignan derivatives. Abbreviations: RT, retention time; *m*/*z*, mass-to-charge ratio.

No	RT (min)	Tentative Identification	[M-H]^−^ (*m*/*z*)	[M-H]^+^ (*m*/*z*)	Error (ppm)	Molecular Formula	Family	MS/MS (*m*/*z*, Relative Abundance)	Ref
1	1.672	4-(methylamino)phenol		124.0756	−4.3	C_7_H_9_NO	Benzoic acids	80.04 (65), 96.04 (60), 108.04 (100)	Database
2 *	2.218	protocatechuic acid	153.0204		3.9	C_7_H_6_O_4_	Benzoic acids	78.98 (52), 108.02 (45), 109.03 (100)	[27,47]
3	2.690	3-O-caffeoylquinic acid (neochlorogenic acid)	353.0890		2.0	C_16_H_18_O_9_	Hydroxycinnamic acids	179.04 (100), 191.06 (69)	[52,53,54]
4 *	3.532	5-O-caffeoylquinic acid (chlorogenic acid)	353.0896		3.5	C_16_H_18_O_9_	Hydroxycinnamic acids	135.05 (1), 173.05 (6), 191.06 (100), 179.04 (1)	[53,54,55]
5	3.841	methyl-3-O-caffeoylquinic acid	367.1049		2.5	C_17_H_20_O_9_	Hydroxycinnamic acids	161.03 (100), 173.05 (7)	[55,56]
6	3.846	2-hydroxy-5-methoxybenzaldehyde	151.0410		3.1	C_8_H_8_O_3_	Hydroxyphenols	92.03 (51), 107.06 (100), 136.02 (82)	Database
7	4.060	cis-ethyl-3-O-caffeoylquinic acid	381.1208		3.1	C_18_H_22_O_9_	Hydroxycinnamic acids	133.03 (19), 161.03 (100), 179.04 (7)	Manual MS/MS interpretation (literature-based)
8	4.441	methyl-4-O-caffeoylquinic acid	367.1052		3.2	C_17_H_20_O_9_	Hydroxycinnamic acids	133.03 (14), 135.04 (19), 161.03 (100)	[55,57]
9	4.446	3-O-caffeoylquinic acid lactone	335.0791		4.0	C_16_H_16_O_8_	Hydroxycinnamic acids	133.03 (5), 135.05 (18), 161.03 (100), 173.05 (6), 179.04 (6)	[43,58]
10	4.616	4-O-caffeoylquinic acid lactone	335.0786		2.6	C_16_H_16_O_8_	Hydroxycinnamic acids	133.03 (6), 135.05 (8), 161.03 (100), 179.04 (5)	[50,51]
11	4.737	ethyl-3-O-caffeoylquinic acid	381.1208		2.9	C_18_H_22_O_9_	Hydroxycinnamic acids	133.03 (3), 135.04 (3), 161.03 (100), 179.03 (7)	Manual MS/MS interpretation (literature-based)
12	4.860	methyl-5-O-caffeoylquinic acid	367.1054		−1.5	C_17_H_20_O_9_	Hydroxycinnamic acids	133.03 (14), 135.05 (32), 161.02 (13), 179.04 (100), 191.06 (3)	[55,57]
13	5.098	5-O-caffeoylquinic acid lactone	335.0788		3.2	C_16_H_16_O_8_	Hydroxycinnamic acids	133.03 (9), 135.04 (4), 161.03 (100), 173.05 (15)	[59,60]
14	5.262	ethyl-4-O-caffeoylquinic acid	381.1206		2.6	C_18_H_22_O_9_	Hydroxycinnamic acids	133.03 (4), 135.05 (22), 161.03 (100), 179.03 (8),	Manual MS/MS interpretation (literature-based)
15	5.416	luteolin 7-O-rutinoside (scolymoside)	593.1523		0.9	C_27_H_30_O_15_	Flavonoids-O-glycosides	285.04 (100), 286.04 (32)	[52,61,62]
16 *	5.506	luteolin-7-O-glucoside (cynaroside)	447.0943		1.0	C_21_H_20_O_11_	Flavonoid-O-glycosides	284.03 (17), 285.04 (100), 286.04 (15)	[29,62,63]
17	5.625	ethyl-5-O-caffeoylquinic acid	381.1208		3.2	C_18_H_22_O_9_	Hydroxycinnamic acids	135.05 (51), 161.03 (29), 179.04 (100), 191.06 (6)	Manual MS/MS interpretation (literature-based)
18	5.644	3,4-dicaffeoylquinic acid	515.1209		2.7	C_25_H_24_O_12_	Hydroxycinnamic acids	173.05 (44), 179.04 (39), 335.08 (28), 353.09 (100)	[54,64]
19	5.745	pinoresinol hexoside	519.1883		1.1	C_26_H_32_O_11_	Lignan derivates	121.94 (8), 151.04 (32), 357.13 (100), 358.14 (45)	[29,62,65]
20	5.762	3,5-dicaffeoylquinic acid	515.1213		3.4	C_25_H_24_O_12_	Hydroxycinnamic acids	135.05 (2), 191.06 (100), 179.04 (23), 353.09 (53)	[60,62,66]
21	5.921	apigenin 7-O-rutinoside	577.1565		−0.5	C_27_H_30_O_14_	Flavonoids-O-glycosides	269.05 (100), 270.05 (14)	Database
22 *	6.072	apigenin 7-O-glucoside	431.0996		1.7	C_21_H_20_O_10_	Flavonoid-O-glycosides	268.04 (100), 269.05 (34)	[52,62]
23	6.091	methyl-3,4-dicaffeoylquinic acid	529.1364		1.4	C_26_H_26_O_12_	Hydroxycinnamic acids	161.02 (73), 163.03 (51), 179.04 (29), 367.11 (100)	[57,60]
24	6.133	4,5-dicaffeoylquinic acid	515.1208		2.5	C_25_H_24_O_12_	Hydroxycinnamic acids	173.05 (85), 191.06 (52), 353.09 (100)	[63,67,68]
25	6.497	methyl-1,4-dicaffeoylquinic acid	529.1361		0.8	C_26_H_26_O_12_	Hydroxycinnamic acids	161.03 (100), 179.03 (15), 191.06 (9), 367.10 (95)	[57,69]
26	6.8772	methyl-3,5-dicaffeoylquinic acid	529.1363		1.3	C_26_H_26_O_12_	Hydroxycinnamic acids	161.02 (58), 179.04 (57), 367.10 (100)	[49,62]
27 *	7.190	ethyl caffeate	207.0678		1.7	C_11_H_12_O_4_	Hydroxycinnamic acids	133.03 (70), 134.04 (78), 135.05 (100), 161.03 (52), 179.04 (20)	[70,71]
28	7.232	ethyl-3,5-dicaffeoylquinic acid	543.1516		0.5	C_27_H_28_O_12_	Hydroxycinnamic acids	161.03 (100), 381.12 (49)	Manual MS/MS interpretation (literature-based)
29	7.250	methyl-1,5-dicaffeoylquinic acid	529.1361		0.8	C_26_H_26_O_12_	Hydroxycinnamic acids	161.02 (57), 179.04 (100), 367.10 (67)	[57,59]
30	7.292	3,5-dicaffeoylquinic acid lactone	497.1106		2.3	C_25_H_22_O_11_	Hydroxycinnamic acids	135.04 (15), 161.03 (69), 179.04 (15), 335.08 (100),	Manual MS/MS interpretation (literature-based)
31 *	7.300	luteolin	285.0419		3.2	C_15_H_10_O_6_	Flavones	133.03 (92), 151.00 (100)	[29,52]
32	7.529	ethyl-1,5-dicaffeoylquinic acid	543.1515		0.4	C_27_H_28_O_12_	Hydroxycinnamic acids	161.03 (100), 179.04 (44), 381.11 (81)	Manual MS/MS interpretation (literature-based)
33	7.592	methyl-4,5-dicaffeoylquinic acid	529.1362		1.0	C_26_H_26_O_12_	Hydroxycinnamic acids	161.03 (100), 179.03 (13), 367.10 (46)	[57,59,69]
34	7.734	1,5-dicaffeoylquinic acid lactone	497.1103		1.7	C_25_H_22_O_11_	Hydroxycinnamic acids	161.03 (17), 179.04 (61), 335.08 (100)	Manual MS/MS interpretation (literature-based)
35	7.933	ethyl-4,5-dicaffeoylquinic acid	543.1514		0.2	C_27_H_28_O_12_	Hydroxycinnamic acids	135.05 (47), 161.03 (74), 179.04 (66), 381.12 (100)	Manual MS/MS interpretation (literature-based)
36 *	8.075	apigenin	269.0473		0.4	C_15_H_10_O_5_	Flavones	117.03 (100), 149.03 (64), 151.00 (83), 201.06 (19), 225.06 (15)	[52]
37	8.100	4,5-dicaffeoylquinic acid lactone	497.1100		1.2	C_25_H_22_O_11_	Hydroxycinnamic acids	161.03 (88), 335.08 (100)	Manual MS/MS interpretation (literature-based)
38	8.944	luteolin 7,3′-dimethyl ether		315.0862	−1.2	C_17_H_14_O_6_	Flavones	300.06 (100), 301.06 (14), 272.07 (6)	[71,72]
39	9.437	4,7-dimethoxyflavonol		299.0922	0.6	C_17_H_14_O_5_	Flavanols	256.07 (100), 284.07 (94)	Database

Asterisks (*) denote compounds confirmed using standards.

The following section focuses on the discussion of the identified metabolites in ABP obtained from PLE under optimized conditions.

#### 2.4.1. Benzoic Acid and Derivatives (Compounds **1**, **2**, **6**)

In detail, compound **1** was identified in ESI (+) mode as 4-(methylamino) phenol (C_7_H_9_NO), providing a pseudo-molecular ion [M+H]^+^ at *m*/*z* of 124.0756 and by comparing the generated MS/MS fragments at *m*/*z* 108.04, 80.04 and 96.04 with the MS/MS spectral database. Compound **2** was confirmed as protocatechuic acid (C_7_H_6_O_4_) and it has been reported in previous studies on artichoke leaves’ extracts [45,48]. Compound **6** was identified as 2-hydroxy-5-methoxybenzaldehyde (C_8_H_8_O_3_), presenting a parent ion [M-H]^−^ at *m*/*z* 151.0410 and producing a MS/MS fragment at *m*/*z* 136.02, confirmed by spectral database comparison.

#### 2.4.2. Hydroxycinnamic Acids Derivatives (Compounds **3**, **4**, **5**, **8**, **12**, **18**, **20**, **23**, **24**, **25**, **26**, **27**, **29**, **33**)

These identified compounds belong to the class of hydroxycinnamic acids, such as mono-caffeoylquinic acids (CQAs) and di-caffeoylquinic acids (di-CQAs), found also in their methyl ester forms. Compounds **3** and **4** (C_16_H_18_O_9_) were identified as 3-CQA and 5-CQA (chlorogenic acid), respectively. Both compounds present similar MS/MS product ions at *m*/*z* 191.05 [quinic acid-H]^−^, 179.03 [caffeic acid-H]^−^ and 135.04 [caffeic acid-CO_2_-H]^−^, but they exhibit slight differences in signal relative intensities. In addition to the different elution order, discrimination between 3-CQA and 5-CQA was possible by observing a higher intensity of the ion at *m*/*z* 179.03 [caffeic acid-H]^−^ for 3-CQA, as described in previous works [47,48,49,50]. According to Clifford’s hierarchical schemes, MSn fragmentation patterns of mono-CQA and di-CQA are strongly influenced by the specific stereochemical arrangements of each substituent on the quinic acid moiety [73]. It was also possible to discriminate among different naturally occurring methyl ester derivatives of CQA (C_17_H_20_O_9_), such as methyl 3-CQA (**5**) (*m*/*z* at 367.1049), methyl 4-CQA (**8**) (*m*/*z* at 367.1052) and methyl 5-CQA (**12**) (*m*/*z* at 367.1054). Specifically, methyl 3-CQA (**5**) and methyl 4-CQA (**8**) provided an MS/MS base peak at *m*/*z* 161.03 [caffeic acid-H_2_O-H]^−^, and the elution order was considered for their discrimination according to the literature data [55,57]. In addition, it was also possible to differentiate the methyl 5-CQA (**12**) according to the fragmentation pattern, providing a characteristic MS/MS base peak at *m*/*z* 179.04, not observed for the other isomers [54,55].

Compounds **18**, **20** and **24** were identified as 3,4-di-CQA, 3,5-di-CQA (isochlorogenic acid A) and 4,5-di-CQA (C_25_H_24_O_12_), providing *m*/*z* at 515.1209 [M-H]^−^, 515.1213 [M-H]^−^ and 515.1208 [M-H]^−^, respectively. These identifications were based on the elution order [59] and the different MS/MS fragmentation patterns. In the case of 3,4-diCQA (**18**), it was identified based on the presence of MS/MS fragments at *m*/*z* 353.09 [CQA-H]^−^, 173.05 [quinic acid-H_2_O-H]^−^, and 179.04 [caffeic acid-H]^−^, and the characteristic ion at *m*/*z* 335.08 [CQA-H_2_O-H]^−^ [73]. In the case of 3,5-diCQA (**20**), it was identified based on the MS/MS base peak at *m*/*z* 191.06 [quinic acid-H]^−^, followed by 353.09 [CQA-H]^−^ and 179.04 [caffeic acid−H]^−^ [54,73]. Finally, 4,5-diCQA (**24**) was identified based on the produced MS/MS fragments at *m*/*z* 353.09 [CQA-H]^−^, 173.05 [quinic acid-H_2_O-H]^−^, 191.06 [quinic acid-H]^−^, and the characteristic ion at *m*/*z* 93.04 [15].

Compounds **23**, **25**, **26**, **29** and **33** were identified as methyl 3,4-, 1,4-, 3,5-, 1,5- and 4,5-CQAs methyl esters (C_26_H_26_O_12_), providing *m*/*z* at 529.1264 [M-H]^−^, 529.1261 [M-H]^−^, 529.1263 [M-H]^−^, 529.1261 [M-H]^−^ and 529.1262 [M-H]^−^, respectively. These identifications were based on the elution order and the MS/MS fragmentation patterns previously reported, and considering the elution order and the abundance observed for the different methyl esters of mono CQAs (compounds **5**, **8** and **12**). In addition, these compounds were differentiated from caffeoyl-feruloylquinic acids as methyl di-CQAs produce MS/MS base peaks at *m*/*z* 161.03 and 179.04, whereas caffeoyl-feruloylquinic acids produce ions at *m*/*z* 353.09 and 335.08 [54,68]. The methyl 3,4-diCQA isomer (**23**) was characterized by a MS/MS base peak at *m*/*z* 367.11 and a prominent ion at *m*/*z* 161.02, while the least abundant isomer, methyl 1,4-diCQA (**25**), was characterized by a MS/MS base peak at *m*/*z* 161.03, followed by the ion at *m*/*z* 367.10. On the other hand, the methyl 3,5-diCQA isomer (**26**) was the most abundant and was characterized by a MS/MS base peak at *m*/*z* 367.11, and two prominent ions at *m*/*z* 161.02 and 179.04; while the methyl 1,5-diCQA isomer (**29**) was characterized by a MS/MS base peak at *m*/*z* 179.04 followed by ions at *m*/*z* 367.10 and 161.02. Finally, the methyl 4,5-diCQA isomer (**33**) was characterized by an MS/MS base peak at *m*/*z* 161.03, followed by the ion at *m*/*z* 367.10 and the ion at *m*/*z* 179.03. Additionally, compound **27** was confirmed as ethyl caffeate (C_11_H_12_O_4_), producing MS/MS fragments at *m*/*z* 179.04 [caffeic acid-H]^−^, 161.03 [caffeic acid-H_2_O-H]^−^, and 135.05 [caffeic acid-CO_2_-H]^−^ [60]. As highlighted in previous studies, the formation of this compound could be explained by the esterification reactions between chemical groups of carboxylic acids and an alcohol (in our case, EtAc) occurring at high temperatures, which lead to ethyl adducts generation [61].

#### 2.4.3. Novel Discovered (Compounds **7**, **9**, **10**, **11**, **13**, **14**, **17**, **28**, **30**, **32**, **34**, **35**, **37**)

In this section, the identification of compounds found in specific forms, such as ethyl esters and lactones of CQAs and di-CQAs and characterized in ABP extracts, is discussed for the first time. Clifford’s hierarchical schemes were employed for the classification and discrimination of CQAs and di-CQAs derivatives and isomers [67], while representative chromatograms and MS/MS spectra are reported in Figures S3–S7, Supplementary Material. Compounds **7**, **11**, **14** and **17** were identified as ethyl CQAs (C_18_H_22_O_9_), all exhibiting similar pseudo-molecular ions [M-H]^−^ at *m*/*z* 381.1208. As for the methyl CQAs detection, it was possible to reveal four ethyl CQAs isomers, and according to previous findings [55] and similarly to the elution order and the abundance of methyl CQAs isomers, they were assigned as cis ethyl 3-CQA (**7**), ethyl 3-CQA (**11**), ethyl 4-CQA (**14**) and ethyl 5-CQA (**17**). In the same way as for methyl CQAs, cis ethyl 3-CQA (**7**), ethyl 3-CQA (**11**) and ethyl 4-CQA (**14**) provided a MS/MS base peak at *m*/*z* 161.03 [caffeic acid-H_2_O-H]^−^, with very low abundant ions at *m*/*z* 133.03 and 179.04; while ethyl 5-CQA (**17**) produced an MS/MS base peak at *m*/*z* 179.04. In addition to the mono-ethyl CQAs, and in the same way as for the methyl di-CQAs, different ethyl di-CQAs (C_27_H_28_O_12_) were tentatively identified based on three molecular ions [M-H]^−^ at *m*/*z* 543.1516, 543.1515 and 543.1514. Among them, compound **28** was identified as ethyl 3,5-di-CQA, compound **32** was identified as ethyl 1,5-di-CQA and compound **35** was identified as ethyl 4,5-di-CQA. All these compounds provided MS/MS fragment ions at *m*/*z* 381.12 [ethyl CQA-H]^−^ and 161.03 [caffeic acid-H_2_O-H]^−^. In addition, compounds **32** and **35** produced abundant MS/MS ions at *m*/*z* 179.04, characteristic of ethyl 5-CQA. As reported in a previous work, the presence of such ethylated conjugates could be due to the extraction conditions employed, such as high temperature (180 °C) and the composition of the solvent, consisting of a mixture of EtOH and EtAc (10/90 *v*/*v*).

On the other hand, compounds **9**, **10** and **13** were identified as CQA lactones (CQL) (C_16_H_16_O_8_), all of them exhibiting similar molecular ions [M-H]^−^ at *m*/*z* 335.0791, 335.0786 and 335.0788. According to previous findings [54] and applying the same rationale for the elution order and the abundance of methyl and ethyl CQAs isomers, they were assigned as 3-CQL (**9**), 4-CQL (**10**) and 5-CQL (**13**). The three compounds provided an MS/MS base peak at *m*/*z* 161.03 [caffeic acid-H_2_O-H]^−^, with low-abundant ions at *m*/*z* 133.03 and 135.05. In addition, none of them presented an abundant MS/MS fragment at *m*/*z* 173.05, characteristic of 1-CQL [56]. Moreover, compounds **30**, **34** and **37** were identified as isomers of di-caffeoylquinic lactone (di-CQL) by detecting [M-H]^−^ ions at *m*/*z* 497.1106, 497.1103 and 497.1100, respectively, with a proposed molecular formula of C_25_H_22_O_11_. They were assigned as 3,5-di-CQL (**30**), 1,5-di-CQL (**34**) and 4,5-di-CQL (**37**), as all of them presented an MS/MS base peak at *m*/*z* 335.08 [CQL-H]^−^ and similar MS/MS fragment ions to those of the mono-CQL (at *m*/*z* 135.04, 161.03 and 179.04). In addition, the same retention time rationale was applied as for the methyl and ethyl CQAs forms.

To our knowledge, these compounds—CQA lactones (compounds **9**, **10**, **13**), ethyl CQA esters (compounds **7**, **11**, **14**, **17**), di-caffeoylquinic lactones (compounds **30**, **34**, **37**), and ethyl di-CQA esters (compounds **28**, **32**, **35**)—represent the first report of their identification in ABP extracts. The formation of these mono- and di-CQLs in the ABP extract is attributable to the specific PLE conditions employed: the high temperature (180 °C) promotes lactone ring closure of chlorogenic acids, a process previously reported in roasted coffee [43], while the presence of EtOH and EtAc in the solvent mixture facilitates esterification reactions at elevated temperatures, generating ethyl adducts analogous to those reported in other polyphenol-rich matrices [61]. It should be noted that these transformation products may differ from their parent compounds in terms of polarity, metabolic fate, and biological activity. While some CQA derivatives—including ethyl caffeate, confirmed here with an authentic standard—have demonstrated independent bioactivity [70], the pharmacological relevance and bioavailability of the full suite of newly identified derivatives remain to be established.

#### 2.4.4. Flavonoid Derivatives (Compounds **15**, **16**, **21**, **22**, **31**, **36**, **38**, **39**)

Apart from the previous hydroxycinnamic acid derivatives, different flavonoids in their free (aglycones), methylated, and glycosylated forms were identified in ABP extract. For instance, compound **31** was confirmed as luteolin (C_15_H_10_O_6_), and it has previously been reported in artichoke extract samples [29,54,63]. Another highly reported flavonoid in artichoke samples and confirmed with a standard was apigenin (**36**). Compound **38** was tentatively identified as luteolin 7,3′-dimethyl ether (C_17_H_14_O_6_), showing a precursor ion [M+H]^+^ at *m*/*z* 315.0862 and by database comparison of the MS/MS fragment ions at *m*/*z* 272.07, 300.06 and 301.06, and compound **39** was identified as 4,7-dimethoxyflavonol (C_17_H_14_O_5_) by database comparison, presenting a precursor ion [M+H]^+^ at *m*/*z* 299.0922 and generating MS/MS fragmentation ions at *m*/*z* 256.07 and 284.07.

Moreover, compounds **15**, **16**, **21** and **22** were identified as flavonoid glycoside derivatives. Compound **15** was tentatively identified as luteolin-7-O-rutinoside (C_27_H_30_O_15_), and compound **16** was confirmed as luteolin-7-O-glucoside (C_21_H_20_O_11_). Both compounds provided a characteristic MS/MS base peak ion at *m*/*z* 285.04 [luteolin-H]^−^, and they have been previously reported in artichoke samples [52]. Similarly, compound **21** was tentatively identified as apigenin-7-O-rutinoside (C_27_H_30_O_14_) and compound **22** was confirmed as apigenin-7-O-glucoside (C_21_H_20_O_10_). Both compounds provided the characteristic MS/MS ion at *m*/*z* 269.05 [apigenin-H]^−^ and they have also been identified in artichoke samples.

#### 2.4.5. Lignan Derivatives (Compound **19**)

Compound **19** was tentatively identified as pinoresinol hexoside (C_26_H_32_O_11_), indicated by the precursor ion [M-H]^−^ at *m*/*z* 519.1883 and MS/MS fragment ions at *m*/*z* 357.13 [pinoresinol-H]^−^, 151.04 and 121.94, which is already reported in the literature [45,60,63].

In summary, most of the compounds identified in the PLE optimum extract obtained from ABP have been previously described in artichokes [42,62,74,75] and their industrial by-products [29,40,45,76]. Nonetheless, this study reports novel identified compounds in ABP for the first time, including caffeoylquinic acid derivatives such as lactone derivatives and ethyl esters. These compounds could be produced during the extraction process, which involves high temperatures and pressures. It has been reported that elevated temperatures can improve the extraction of phenolic acids [72] and free flavonoids, or generate new adducts—such as melanoidins—with higher antioxidant potential [38,39]. Furthermore, since EtAc was used as the extraction solvent, formation of ethylated adducts, likely promoted by the used PLE conditions, can occur.

### 2.5. Blood–Brain Barrier (BBB) In Vitro Permeability Evaluation of ABP Optimal Extract

The blood–brain barrier (BBB) acts as a selective and dynamic interface protecting the central nervous system (CNS), regulating the passage of molecules between the bloodstream and brain tissue. Its function is critical for maintaining CNS homeostasis, yet it poses a major challenge for drug delivery to the brain. The ability of bioactive compounds to cross the BBB is essential for achieving neurotherapeutic efficacy. Molecules such as cholinesterase and lipoxygenase inhibitors rely on favorable physicochemical characteristics—particularly molecular weight (M_W_), lipophilicity (LogP), and topological polar surface area (TPSA)—to effectively reach their targets in brain tissue. Evidence from prior studies [13,77] indicates that compounds with M_W_ < 500 Da, LogP between 0 and 3, and TPSA < 90 Å^2^ show enhanced BBB permeability. For instance, researchers reported the detection of low-molecular-weight phenolic acids such as protocatechuic acid in the brains of mice following oral administration [78], highlighting the potential for natural compounds from agro-industrial by-products to cross the BBB and exert neuroprotective effects. Indeed, the neuroprotective activity of flavonoid aglycones such as apigenin and luteolin has been extensively documented in both in vitro neuronal models and in vivo rodent studies, with mechanisms involving AChE/BChE inhibition, modulation of oxidative stress pathways, and attenuation of neuroinflammation [68,79].

In this study, an in vitro parallel artificial membrane permeability assay (PAMPA-BBB) was used to evaluate the capacity of bioactive compounds present in artichoke by-product (ABP) extracts to cross the BBB. The permeability performance was assessed using experimental permeability coefficients (Log Pe), alongside LogP values (from PubChem) and TPSA. Table 4 shows the experimental permeability coefficients (Log Pe, calculated as described in Section 3.12), lipophilicity values (LogP, obtained from PubChem), and the chemical families of compounds able to cross the artificial BBB.

**Figure 3:**
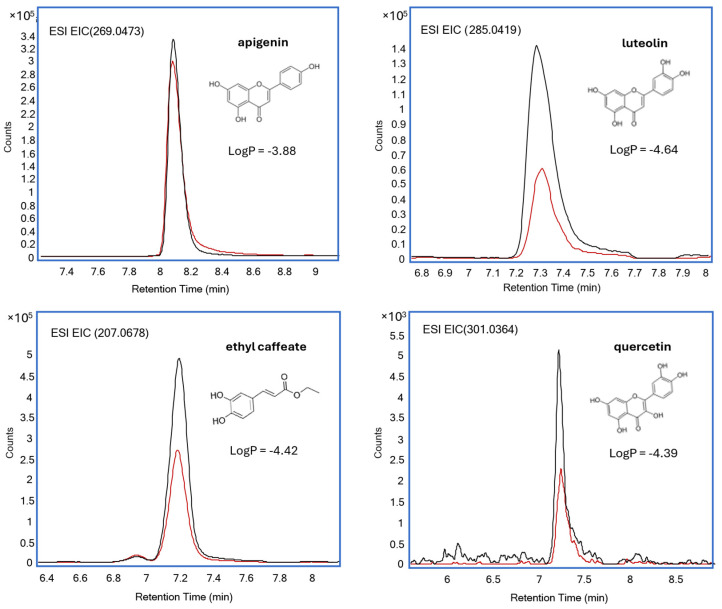
Representative extracted ion chromatograms of bioactive compounds identified in ABP PLE extracts obtained under optimized conditions. Black lines represent signals from the donor plate, while red lines correspond to the acceptor plate in the in vitro PAMPA-BBB assay.

Based on these outcomes, the highest Log(Pe) values (<4.50) for ABP PLE extracts were primarily related to phenolic compounds, such as protocatechuic acid and ethyl caffeate, as well as flavonoids like apigenin and luteolin. These findings highlight their potential to cross the BBB and contribute to neuroprotective effects. For example, bioactive compounds such as protocatechuic acid (LogP = 0.40) and luteolin (LogP = 2.12) showed high permeability despite differences in their chemical structures and degree of lipophilicity. In this regard, LogP values between 0 and 3 have been reported to have an enhanced ability to cross the BBB [11]. Molecular weight (M_W_) is another key parameter influencing the diffusion performance of various biomolecules. Previous research has shown that compounds with an M_W_ below 500 Da can effectively cross the BBB [38]. In our case, compounds such as apigenin (M_W_ = 270.24 Da) and ethyl caffeate (M_W_ = 194.18 Da) found in ABP gave high experimental diffusion values (Log(Pe) = −3.88 and Log(Pe) = −4.42, respectively). Larger molecules, such as di-caffeoylquinic acids (di-CQAs) or glycosylated flavonoids, showed limited permeability, likely due to their larger molecular size and higher polarity, as previously reported [11]. Topological polar surface area (TPSA) also plays a critical role in diffusion across the BBB. It is generally observed that compounds having TPSA below 90 Å^2^ tend to diffuse better across the BBB [11,38]. Flavonoids such as apigenin (TPSA = 87 Å^2^) provided the highest diffusion performance in terms of Log(Pe). On the other hand, glycosylated compounds such as apigenin-7-rutinoside, characterized by a higher TPSA (225 Å^2^), showed markedly reduced ability, which is consistent with its high polarity and large molecular size. In Figure 3, representative extracted ion chromatograms of the bioactive compounds identified in ABP PLE optimal extract after performing the PAMPA-BBB assay are displayed.

Interestingly, bioactive molecules such as apigenin, ethyl caffeate, and other phenolic compounds derived from artichoke industrial by-products have demonstrated the ability to cross the blood–brain barrier, effectively reaching their targets for the treatment of neurodegenerative diseases. Their therapeutic potential is further supported by their favorable physicochemical properties, including optimal molecular weight, balanced lipophilicity (LogP), and efficient diffusion rates, positioning them as promising candidates for drug development.

### 2.6. Molecular Docking Simulations

**Figure 4:**
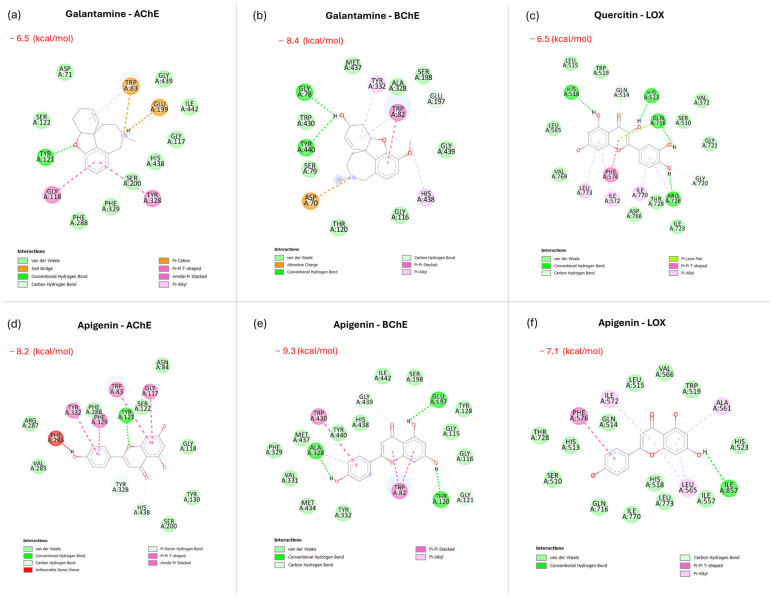
Molecular docking simulations of Acetylcholinesterase (AChE), Butyrylcholinesterase (BChE) and Lipoxygenase (LOX) with corresponding positive controls (galantamine for cholinergic enzymes, quercitine for LOX) and apigenin identified in ABP PLE extract. (**a**) AChE-galantamine; (**b**) BChE-galantamine; (**c**) LOX-quercetin; (**d**) AChE-apigenin; (**e**) BChE-apigenin; (**f**) LOX-apigenin.

The molecular docking approach is an effective tool to identify specific ligands by predicting their binding modes within the target protein. In this study, docking simulations were performed to investigate the binding affinity scores of three enzyme complexes—AChE, BChE, and LOX—and apigenin, one of the most abundant flavonoids identified in ABP PLE extract obtained under optimized conditions. The analyses revealed several possible complexes based on the different flavonoid orientations within the enzyme active sites. Figure 4 presents a comparative analysis of docking complexes and binding affinity scores involving positive controls (galantamine for cholinergic enzymes and quercetin for LOX) and apigenin.

In silico docking simulations revealed favorable binding modes for apigenin within the active sites of all three enzymes, providing a mechanistic hypothesis for the observed inhibitory activity. It is important to emphasize that docking binding affinity scores are calculated for a single pure compound interacting with an isolated enzyme in silico and therefore cannot be directly compared to, or used to predict, the IC_50_ values of a complex crude extract. The apparent discrepancy between the favorable in silico binding affinities and the moderate in vitro IC_50_ values is therefore expected and mechanistically consistent: in the crude extract, apigenin constitutes only one of 39 identified compounds, and its effective concentration at the enzyme active site is substantially diluted by the matrix. Comparing the docking scores of apigenin (AChE: −8.2 kcal/mol) and galantamine, the simulations confirm that apigenin can occupy and interact with the AChE catalytic gorge in a sterically compatible manner, consistent with previous reports on flavonoid-enzyme binding [80].

In general, docking analysis highlighted hydrogen bond interactions with the residual amino acid TYR121 (peripheral anionic site) and π–π stacking interactions with TRP83 and TYR332, consistent with previous reports on flavonoid-enzyme binding behavior [80]. Additionally, unfavorable bonds were observed between PHE286 and the B-ring hydroxyl group, which may impact binding stability. These structural features suggested that apigenin interacts more effectively with cholinergic enzymes, in agreement with prior findings on its selective binding and inhibitory capacity [68]. For the BChE-apigenin complex, the docking simulations revealed an even higher binding score (−9.3 kcal/mol), surpassing the binding affinity of its positive control. This indicates a strong interaction of apigenin with the BChE active site. The differences in binding affinity scores between AChE and BChE could be attributed to the differences in the deep gorge structure of the two cholinergic enzymes. Notably, the BChE catalytic site contains fewer hydrophobic aromatic amino acid residues, allowing better interactions with relatively polar ligands such as apigenin [27,46]. Hydrogen bonds primarily mediated these interactions, facilitated by the hydroxyl groups of flavonoids and the amino acid residues of the BChE catalytic site (GLY115, TYR128, and GLU197).

For LOX complexes, docking simulations involving apigenin revealed comparable binding affinity scores, as this flavonoid shares structural similarities with the chemical structure of quercetin (positive control). Specifically, the docking analysis showed six conformers with binding affinities ranging from −4.8 to −7.1 kcal/mol. The binding modes were assessed by replacing the natural co-crystallized ligand with apigenin. The reference compound, quercetin, exhibited a high binding score (−7.1 kcal/mol). Consistent with previous findings, docking simulations confirmed that quercetin interacts with the LOX active site through hydrogen bonding with His, Gln, and Arg residues. It was demonstrated that apigenin fits into the active sites and forms binding interactions in a similar way. It also forms multiple weak interactions with key amino acids (ALA561, LEU565, ILE572 and PHE576). Additionally, a hydrogen bond was observed between ILE857 and the hydroxyl groups of its bicyclic ring, which further stabilizes the enzyme-inhibitor complex.

Taken together, the docking results support the plausibility of apigenin as a contributing active agent within the extract but should be interpreted as mechanistically hypothesis-generating rather than predictive of extract potency. The moderate IC_50_ values observed in the enzyme inhibition assays (AChE: 523 µg/mL; BChE: 405 µg/mL; LOX: 275 µg/mL) reflect the inherent characteristics of a complex crude extract evaluated against highly purified enzyme preparations and are broadly consistent with values reported for comparable agro-industrial by-product extracts in the literature [81,82,83]. Factors contributing to the attenuation of activity in the crude extract relative to pure compound predictions include: (i) dilution of active constituents within the total extract mass, (ii) matrix effects and potential competitive or antagonistic interactions among co-extracted compounds [84], and (iii) possible degradation or structural modification of sensitive bioactive molecules under the high-temperature extraction conditions employed. The IC_50_ values measured here therefore characterize the ABP PLE extract as a source of moderate neuroprotective and anti-inflammatory activity, consistent with its nature as a complex agro-industrial by-product extract rather than a purified pharmaceutical preparation [85].

## 3. Materials and Methods

### 3.1. Solvents, Reagents and Standards

Milli-Q water was collected from the Millipore system (Billerica, MA, USA). Absolute ethanol (EtOH), ethyl acetate (EtAc), HPLC-grade acetonitrile (ACN), cyclopentyl methyl ether (CPME), and HPLC-grade methanol were supplied by VWR Chemicals (Barcelona, Spain). For conducting in vitro biological activity assays, sodium chloride, sodium acetate, sodium hydroxide, sodium dihydrogen phosphate, potassium phosphate, and monopotassium phosphate were obtained from Panreac Quimica SLU (Barcelona, Spain). The Folin–Ciocalteu reagent was acquired from Merck (Darmstadt, Germany). Trizma hydrochloride (Tris-HCl), AChE Type VI-S derived from *Electrophorus electricus*, BChE from equine serum, linoleic acid (LA), acetylthiocholine iodide (AChI), aluminum chloride (AlCl_3_), phosphoric acid, sodium carbonate, potassium phosphate, monopotassium phosphate, fluorescein, gallic acid, quercetin, galantamine hydrobromide, and a 96-well acceptor plate were sourced from Sigma-Aldrich (Madrid, Spain). Additionally, LOX from *Glycine max* (soybean), 4-(amino-359-sulfonyl)-7-fluoro-2,1,3-benzoxadiazole (ABD-F), and 2,2-azobis(2-amidinopropane) dihydrochloride (AAPH) were purchased from TCI Chemicals (Tokyo, Japan). Luteolin 7-O-glucoside was purchased from Extrasynthese (Genay, France), while protocatechuic acid and chlorogenic acid were obtained from Sigma-Aldrich (St. Louis, MO, USA). Apigenin 7-O-glucoside, luteolin, apigenin and ethyl caffeate were acquired from BLD Pharmatech (Reinbek, Germany).

### 3.2. Artichoke By-Product (ABP) Samples

**Figure 5:**
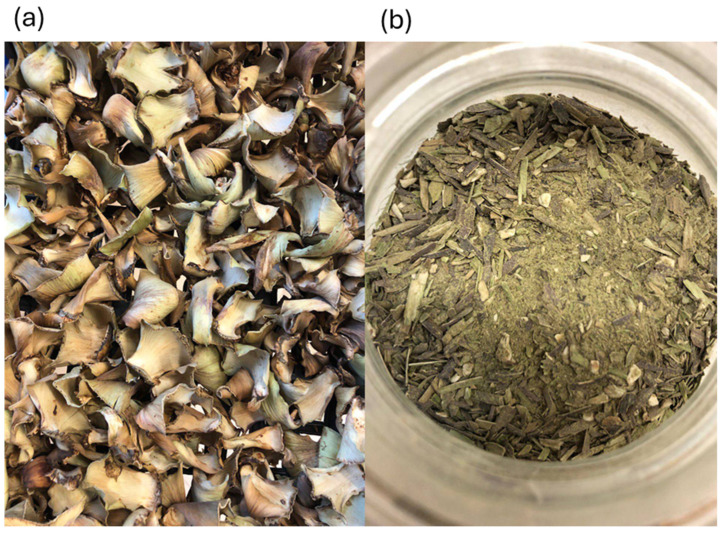
Representative images of artichoke by-product (ABP) bracts at different processing stages: (**a**) freshly dried bracts after oven-drying at 40 °C; (**b**) ground bracts after milling and sieving (300–600 µm particle size).

Artichoke by-product (ABP) samples, consisting mainly of outer bracts derived from the industrial processing of artichoke heads, were supplied by Greci Industria Alimentare S.p.A (Ravadese, Parma, Italy), from 2023 harvesting. As the predominant waste fraction, outer bracts were collected after processing artichoke-based products and used as secondary raw materials. They were oven-dried at 40 °C until reaching constant dry weight. Then, dried samples were finely ground using a laboratory-grade knife mill Grindomix GM 200 (Retsch GmbH, Haan, Germany) and sieved to obtain a uniform particle size (300–600 μm) (see Figure 5). Lastly, the resulting powder was vacuum-packed (C400 Multivac Wolfertschwenden, Germany) and stored at −18 °C.

The chemical composition of ABP can vary with genotype, origin, harvest season, processing degree, and post-harvest handling [1,19]. This work used a single batch from one supplier, which limits the assessment of batch-to-batch variability. Although the standardized pre-treatment (drying, milling, sieving) reduced within-batch heterogeneity, future work should evaluate inter-batch and inter-cultivar variability in both composition and biological activity to confirm the robustness and transferability of the optimized PLE protocol.

### 3.3. Pressurized Liquid Extraction (PLE) Method

Pressurized liquid extraction (PLE) of bioactive compounds from ABP was performed using an ASE 200 (Dionex, Sunnyvale, CA, USA) instrument, equipped with a solvent controller. In a first optimization step, four solvents with different polarities (water, EtOH, EtAc and CPME) were used and the PLE parameters selected—such as pressure, temperature, number of cycles and extraction time—were set at 10.34 MPa, 115 °C, 1 cycle and 20 min, respectively. To perform the extraction, 1 g of ABP powder was placed into a 5 mL extraction cell and mixed with 2 g of sand which was used as supporting material. The cell was filled with the selected solvent and then two cellulose filters were placed on the top and bottom of the cell (Restek, Bellefonte, PA, USA). The extracts were collected in a glass vial (final volume of 17 mL) and dried using a nitrogen solvent evaporator at 30 °C (TurboVap LV, Caliper LifeSciences, Hopkinton, MA, USA). Sample extracts were stored at −18 °C until further use.

### 3.4. Optimization of PLE Conditions by Response Surface Methodology (RSM)

Based on the results obtained in the preliminary test (see Section 2.3), EtOH and EtAc solvents were selected and a response surface methodology (RSM) based on a central composite design (CCD) approach was employed to further optimize the PLE conditions to maximize the bioactive compound recovery. Two factors were then considered at three levels: X1 as percentage of EtOH in EtAc (%, *v*/*v*) and X2 as extraction temperature (°C). Statgraphics Centurion XVI software (v.16.1.11) (StatPoint Technologies, Inc., Warrenton, VA, USA) was employed to design eleven experiments comprising a full factorial design with four runs, four axial (star) points, and three replicates at the central point, assessing the selected response variables (Yi): extraction yield (%), TPC (as mg of gallic acid equivalents, GAE/g dry extract), TFC (as mg of quercetin equivalents, QE/g dry extract), ORAC and DPPH assays (both expressed as IC_50_, μg/mL). The proposed quadratic model (Equation (1)) for each response variable was:

(1) $$Y_{i}=a+X_{1}+X_{2}+X_{1}X_{2}+X_{1}^{2}+X_{2}^{2}+\varepsilon$$

The experimental design was set as follows: the independent variables were *X*_1_ as percentage of EtOH in EtAc (0, 50, and 100%, *v*/*v*), and *X*_2_ as temperature (50, 115, and 180 °C).

### 3.5. Extraction Yield, Total Phenolic Content (TPC), and Total Flavonoid Content (TFC)

The global extraction yield obtained by PLE was calculated as the percentage of the extract mass on a dry basis and the mass of the initial dry sample used. The total extraction yield was calculated as follows (Equation (2)):

(2) $$Extraction\;yield\;\%=\frac{extracted\;mass}{initial\;mass}\times 100$$

TPC and TFC of the extracts obtained by PLE from ABP were determined according to the protocol previously reported [86]. For TPC measurement, 10 μL (concentration 5 mg/mL) of each extract was added to 50 μL of Folin reagent. After 1 min, 150 μL of a 20% (*w*/*v*) aqueous sodium carbonate solution was added and the volume was made up to 1 mL with water. After 2 h of incubation at room temperature in darkness, 300 μL of the mixture was transferred into a micro-well plate. The absorbance of solutions was measured at 760 nm with a Synergy HT microplate reader, by BioTek Instruments (Winooski, VT, USA). The calibration curve was made using gallic acid as a standard in a range between 0.031 and 2 μg/mL in Milli-Q water. The results were expressed as mg GAE/g dry extract. All the analyses were performed in triplicate. For TFC, the measurement was performed according to the AlCl_3_ colorimetric assay as reported above [34]. Briefly, 100 μL of extract (concentration 5 mg/mL) was added to 140 μL of methanol and 60 μL of an 8 mM aqueous solution of AlCl_3_. After 30 min of incubation in the dark, the absorbance was measured at 425 nm. TFC was calculated from a calibration curve using quercetin as a standard in a range from 1 to 14 μg/mL. Results were expressed as mg QE/g dry extract. All the analyses were performed in triplicate.

### 3.6. Oxygen Radical Absorbance Capacity (ORAC)

The oxygen radical absorbance capacity (ORAC) method was used to determine the antioxidant activity by evaluating fluorescence degradation. The method followed was already reported, but with slight modifications [13]. A 96-well microplate in an automated plater fluorimeter reader (BioTek Intruments Inc., Winooski, VT, USA) was used for the analysis. In brief, the reaction mixtures in the wells contained the following reagents: 100 μL of extract sample at different concentrations (500–5000 μg/mL) in EtOH, 100 μL of AAPH (590 mM) in 30 mM phosphate-buffered saline (PBS) at pH = 7.5 were added to generate peroxyl radicals. Before the measurement, extracts were mixed with 25 μL of fluorescein (11 μM). In the microplate reader, fluorescence was measured by the following conditions: λ_excitation_ = 485 nm and λ_emission_ = 530 nm. The absorbances were recorded every 5 min at 37 °C for 1 h. The calibration curve was made using Trolox 15 μM as a standard. The results were expressed as IC_50_ (μg/mL). All the measurements were performed in triplicate.

### 3.7. DPPH Radical Scavenging Activity

The radical scavenging capacity was assessed using the DPPH radical, following a modified protocol [35]. Specifically, each well was filled with varying volumes of the sample, ranging from 10 to 100 μL at a concentration of 1.5 mg/mL. Next, 150 μL of DPPH solution (6 × 10^−5^ M in EtOH) was added to each sample. After incubating in the dark at room temperature for 30 min, absorbance was measured at 517 nm using a microplate reader. The results were expressed as IC_50_ in μg/mL and calculated using linear regression, with concentration as a function of free radical scavenging activity. A solvent mixed with the sample extract served as the blank, while the DPPH solution (150 μL; 6 × 10^−5^ M) was the negative control. All measurements were conducted in triplicate.

### 3.8. Total Chlorophyll Content

For the determination of chlorophyll pigments, the method described by Amador-Luna [87], modified for micro-concentrations, was employed. Absorbance measurements were conducted using a UV-VIS spectrophotometer (Spectronic 200E, version 4.07i, Thermo Fisher Scientific, Mundelein, IL, USA) at wavelengths of 664 nm, 647 nm, and 630 nm for chlorophyll a (Equation (3)), chlorophyll b (Equation (4)), and chlorophyll c (Equation (5)), respectively. Total chlorophyll content was calculated by summing each chlorophyll content (Equation (6)). Turbidity correction was performed by measuring absorbance at 750 nm. Chlorophyll concentrations were calculated using the formulas established by Arnon [88].

(3) $$Chl\;a\;\left(\frac{mg}{L}\right)=12.7\times A_{664}-2.69\times A_{647}$$

(4) $$Chl\;b\;\left(\frac{mg}{L}\right)=22.9\times A_{647}-4.68\times A_{664}$$

(5) $$Chl\;c\;\left(\frac{mg}{L}\right)=24.36\times A_{630}-3.85\times A_{647}$$

(6) Chl Total=Chl a+Chl b+Chl c

### 3.9. Total Carotenoid Content

Total carotenoid contents were measured by colorimetric assays. The extract (0.05 mg/mL) was dissolved in cold acetone/Milli-Q water (90:10, *v*/*v*) after an overnight incubation in the dark and at 4 °C. The mixture was centrifuged at 1500× *g* rpm for 10 min at 4 °C. The supernatant was collected and diluted with 5 mL acetone/Milli-Q water (90:10, *v*/*v*) and the absorbance was measured at 480 and 750 nm. The value of total carotenoid content was obtained with Britton’s [89] formula (Equation (7)).

(7) $$Total\;Carotenoid\;Content\;\left(\%\right)=A\cdot \frac{10^{6}}{A_{1cm}^{1\%}\cdot 0.03\cdot \left[M^{ext}\right]}\cdot 100\%$$

### 3.10. Cholinesterase and Lipoxygenase Inhibitory Assays

The acetylcholinesterase (AChE) and butyrylcholinesterase (BChE) inhibitory activities of ABP PLE extracts were determined according to the fluorescent enzyme kinetic method [13]. Galantamine hydrobromide was dissolved in 50% EtOH and used as the reference inhibitor, 50% EtOH was used as the blank control. Lipoxygenase (LOX) inhibitory capacity of ABP PLE extracts was also determined [11]. Quercetin was used as a reference inhibitory and 50% EtOH was used as a blank control.

### 3.11. HPLC-C18-Q-ToF MS/MS Analysis

The PLE extract of ABP, obtained under optimized conditions, was dissolved in EtOH, reaching a final concentration of 2 mg/mL. The sample was then vortexed for 30 s and centrifuged at 14,800× *g* rpm for 5 min at 4 °C. The supernatant was collected and stored at −80 °C. A volume of 2 μL was then injected into the HPLC system model 12,290 (Agilent Technologies) coupled to the Q-TOF series 6540 using an Agilent Jet Stream thermal orthogonal ESI source (Agilent Technologies, Waldbronn, Germany). Compound separation was performed using a ZORBAX Eclipse Plus C18 analytical column (100 mm × 2.1 mm, 1.8 μm particle size) and a C18 guard column (0.5 cm × 2.1 mm, 1.8 μm particle size) supplied by Agilent Technologies (Waldbronn, Germany). Chromatographic analysis was performed using a gradient elution with Milli-Q water modified with 0.1% formic acid (mobile phase A) and acetonitrile (ACN) with 0.1% formic acid (mobile phase B). The gradient consisted of 0–30% B over 7 min, 30–80% B over 2 min, 80–100% B over 2 min, held at 100% B for 2 min, and a post time of 3 min to return to initial conditions. The column temperature was maintained at 40 °C with a flow rate of 0.5 mL/min. The mass spectrometer was operated in ESI positive (+) and ESI negative (−) modes using the following parameters: *m*/*z* range: 40 to 1700; capillary voltage: 3000 V; fragmentor voltage 110 V; skimmer voltage: 45; octapole voltage 750 V; nebulizer pressure: 40 psig; drying gas flow rate: 8 L/min; drying gas temperature: 300 °C; Sheath gas flow was: 11 L/min; sheath gas temperature: 350 °C. MS/MS analyses were performed using collision energies of 20 and 40 V. To ensure accurate mass measurements, the spectra were calibrated using ions *m*/*z* 121.0509 (C_5_H_4_N_4_) and 922.0098 (C_18_H_18_O_6_N_3_P_3_F_24_) in ESI (+) mode and *m*/*z* 119.0363 (C_5_H_4_N_4_) and 980.0164 (C_18_H_18_O_6_N_3_P_3_F_24_ + acetate) in ESI (−) mode. A blank sample of EtOH was included for blank subtraction. The chromatograms were processed using MS-DIAL v4.9 software for tentative identification of compounds, supplemented by MS/MS spectral comparisons with the NIST, LipidBLAST and MoNA databases. Additionally, Agilent MassHunter Qualitative Analysis software (version B.10.0) was utilized to obtain further qualitative insights into the tentatively identified compounds, employing filtering tools based on diagnostic product ions and/or neutral losses of interest to enhance data interpretation.

### 3.12. Parallel Artificial Membrane Permeability Assay for the Blood–Brain Barrier (PAMPA-BBB)

The PAMPA-BBB in vitro assay was performed in accordance with the protocol described by Sánchez-Martínez [11]. In summary, the BBB solution was prepared by firstly dissolving 8 mg of PBL and 4 mg of cholesterol in 600 μL of n-dodecane. Thereafter, 5 μL of the BBB solution was applied to the filter membrane of the donor plate. Subsequently, 1 mL of extract (10 mg/mL in EtOH) was mixed with 1 mL of buffer solution (PBS, pH = 7.4, 10 mM) to achieve a final volume of 2 mL. The acceptor plate was then filled with 350 μL of buffer, after which the donor plate was carefully assembled over the acceptor plate to create a “sandwich” configuration. Finally, 350 μL of donor solution was added, the plates were sealed, and the incubation period was set at 37 °C and 37% relative humidity. Following the designated incubation time, a volume of 300 μL from both plates was transferred to separate vials and dried in the SpeedVac system at 40 °C and 13 mBar pressure. The dried acceptor and donor solutions were then dissolved in 50 μL of pure EtOH, after which they were analyzed by UHPLC-Q-TOF-MS as described in the previous section. Additionally, supplementary data regarding bioactive compounds identified after PAMPA-BBB, such as LogP (octanol-water partition coefficient) or topological polar surface area (TPSA), were obtained from the PubChem database (https://pubchem.ncbi.nlm.nih.gov/, accessed on 19 February 2024). The in vitro permeability across an artificial BBB is calculated based on the following Equation (8), as previously reported [90]:

(8) $$P_{e}=\frac{-ln\left[1-\frac{C_{A}\left(t\right)}{C_{e}}\right]}{A\times \left(\frac{1}{V_{D}}+\frac{1}{V_{A}}\right)\times t}$$

In this equation, P_e_ refers to the permeability of the bioactive compound across PAMPA-BBB in cm/s; A is the effective filter area, which is given by the manufacturer and is 0.3 cm^2^; V_D_ is the donor well volume, which is 0.35 mL; V_A_ is the acceptor well volume, which is 0.35 mL; t is the incubation time (s) = 14,400; C_A_(t) is the relative area of the compound in acceptor well at time t; and C_D_(t) is the relative area of the compound in donor well at time t. C_equilibrium_ was calculated according to the following Equation (9):

(9) $$C_{equilibrium}=\frac{\left[C_{D}\left(t\right)\times C_{A}\left(t\right)V_{A}\right]}{\left(V_{D}+V_{A}\right)}$$

### 3.13. Molecular Docking

A docking simulation was performed to predict the binding affinity of the most abundant bioactive compound specifically extracted by PLE for the active site of AchE (PDB: 5HFA), BChE (PDB: 6EQP) and LOX (PDB: 1JNQ) enzymes. A single crystal structure of each enzyme was downloaded from the Protein Data Bank (PDB) (http://www.rscb.org/pdb, accessed on 1 February 2024). Apigenin was the most abundant flavonoid in artichoke by-products PLE optimal extract and its structure was obtained from PubChem in SDV format (https://pubchem.ncbi.nlm.nih.gov/, accessed on 1 February 2024). Before docking simulations, AChE, BChE and LOX were pre-processed (dehydrated, hydrogenated and polarized) and then defined as the receptors; while apigenin (CAS: 520-36-5) was defined as the ligand. Both proteins and the ligand were prepared for docking using Chimera software (version 1.14). In the active site of each enzyme, the following XYZ coordinates were used to construct the grid box: X: −2.686, Y: −49.014, Z: 30.162 for AChE; X: 132.836733, Y: 116.056133, Z: 41.709600, for BChE; and X: 27.383, Y: 4.270, Z: 15.298 for LOX. Computational modeling and simulations were conducted using Discovery Studio 4.5 and Materials Studio [24.1.0.0] (Dassault Systèmes BIOVIA, San Diego, CA, USA). Theoretical calculations utilized the Forcite and Discover Studio 4.5, employing both COMPASS and COMPASS-II forcefields (Dassault Systèmes BIOVIA, San Diego, CA, USA) to select the highest free binding energy of the complex ligand-enzyme active site for further analysis using the Lamarckian algorithm.

Finally, the results of the docking simulation for the positive control group (galantamine) were compared with those for apigenin, which showed the best statistical correlation with the enzyme inhibition activity.

### 3.14. Statistical Analysis

Statistics were calculated using GraphPad Prism (v. 6.0; GraphPad Software). Results are expressed as mean ± standard error of the mean (SEM). Statistical comparison between groups was estimated using the one-way analysis of variance (ANOVA), followed by Tukey’s post hoc test. In all cases, *p*-values lower than 0.05 were considered as statistically significant.

## 4. Conclusions

In conclusion, this study demonstrated the potential for sustainable valorization of artichoke by-products (ABP) through a green extraction strategy combining pressurized liquid extraction (PLE) with response surface methodology (RSM). Optimal conditions—using a GRAS solvent mixture of ethyl acetate and ethanol (90:10 *v*/*v*, 180 °C) yielded high recovery of phenolic and flavonoid compounds with antioxidant, neuroprotective, and anti-inflammatory properties. Chemical profiling of the optimized ABP extract revealed a rich composition of phenolic and flavonoid compounds, including novel derivatives such as lactone and ethyl CQA compounds, likely formed during the extraction.

In vitro assays showed moderate inhibition of AChE, BChE, and LOX enzymes, while the PAMPA-BBB assay demonstrated the potential permeability of key compounds across the BBB (e.g., protocatechuic acid, ethyl caffeate, apigenin, luteolin), supporting their neurotherapeutic potential.

However, the chemical complexity of ABP extracts and variability related to cultivar, season, and upstream processing (drying, grinding, storage) pose challenges for standardization, reproducibility, and scalability, requiring systematic validation. The toxicological profile of newly formed compounds (e.g., CQA lactones, ethyl esters) remains unclear and warrants further investigation.

While PLE represents a sustainable approach aligned with green chemistry and circular economy principles, its implementation is constrained by operational costs, including solvent use, energy demand at high temperatures, and equipment investment. As such, it is currently more feasible for large-scale industrial applications than for small- or medium-scale producers. Process optimization (e.g., reduced extraction times, solvent recycling, integration with existing processing streams) could improve economic feasibility and support wider adoption.

Future work should focus on targeted analytical approaches and bioactivity-guided fractionation to identify and absolutely quantify bioactive compounds, alongside the development of standardized protocols to ensure reproducibility. Overall, ABP represents a promising source of bioactive compounds for neuroprotective and anti-inflammatory applications.

## Figures and Tables

**Table 1:** Experimental design and results of response variables (extraction yield, TPC, TFC, ORAC and DPPH) of artichoke by-product (ABP) extracts obtained by PLE. Asterisk (*) refers to the standard used for antioxidant capacity evaluation. Extraction yield calculated as (extract mass/initial dry sample mass) × 100, according to Equation (2).

Run	Temperature	Solvent Composition	Extraction Yield	TPC	TFC	DPPH	ORAC
	°C	EtOH in EtAc (%, *v*/*v*)	mg/g d.e. (%)	mg GAE/g d.e.	mg QE/g. d.e.	IC_50_ (μg/mL)	
1	180	0	7.1	177.3 ± 2.3	10.3 ± 0.4	119.2 ± 2.3	1.5 ± 0.1
2	115	0	5.8	79.2 ± 1.8	8.9 ± 0.2	146.2 ± 3.5	2.1 ± 0.3
3	50	50	3.7	72.1 ± 1.2	4.8 ± 0.1	217.2 ± 1.2	3.6 ± 0.3
4	115	50	5.7	98.4 ± 1.1	7.8 ± 0.6	220.3 ± 2.8	4.2 ± 0.4
5	115	50	7.1	123.4 ± 0.3	6.3 ± 0.5	215.5 ± 3.2	5.3 ± 0.6
6	115	50	6.4	103.6 ± 1.1	7.1 ± 0.6	209.4 ± 3.2	4.6 ± 0.3
7	180	50	9.4	146.3 ± 1.2	9.4 ± 0.4	128.3 ± 1.3	2.0 ± 0.2
8	115	100	9.5	85.9 ± 0.6	5.3 ± 0.4	204.7 ± 1.4	10.7 ± 0.8
9	50	100	7.6	38.1 ± 2.3	6.5 ± 0.3	220.6 ± 2.8	11.7 ± 0.7
10	50	0	3.9	35.3 ± 1.2	7.8 ± 0.1	165.4 ± 1.1	2.5 ± 0.2
11	180	100	12.0	137.3 ± 2.0	4.5 ± 0.3	174.8 ± 1.4	3.3 ± 0.2
Trolox *						4.8 ± 0.2	0.3 ± 0.0

GAE: Gallic acid equivalents; QE: Quercetin equivalents.

**Table 2:** Comparison between predicted and experimental values of the optimal extract obtained under optimized PLE conditions.

Sample	Temperature	Extraction Yield	TPC	TFC	DPPH	ORAC
	°C	%	mg GAE/g d.e.	mg QE/g d.e.	IC_50_, (μg/mL)	
Predicted Opt	180	7.5	166.9	10.4	1.1	119.2
ABP PLE Opt	180	7.7	165.8 ± 1.2	11.2 ± 0.7	1.1 ± 0.1	123.0 ± 1.5

**Table 4:** LogP and experimental PAMPA-BBB values of bioactive compounds identified in ABP extracts obtained under optimized PLE conditions. Row shading indicates chemical family: light blue = benzoic acids and hydroxyphenols; light yellow = hydroxycinnamic acids (mono-caffeoylquinic acid derivatives); light green = flavonoids.

Metabolite Name	[M-H]^−^/[M+H]^+^ *	Family	TPSA	LogP	PAMPA-BBB Log (Pe)	RSD Log (Pe)	Cross BBB Potential ^1^
	(*m*/*z*)		(Å^2^)		(cm/s)	(%)	
apigenin *	269.0473	Flavones	87	2.49	−3.88	0.30	+++
protocatechuic acid *	153.0204	Hydroxybenzoic acid	77.8	0.40	−4.09	0.08	+++
ethyl caffeate *	161.0257	Hydroxycinnamic acids	66.8	1.38	−4.42	0.86	+++
luteolin*	285.0419	Flavones	107	2.12	−4.64	0.91	++
2-hydroxy-5-methoxybenzaldehyde	151.0410	Hydroxyphenols	46.5	1.58	−5.06	0.25	++
5-O-caffeoylquinic acid (neochlorogenic acid)	353.0890	Hydroxycinnamic acids	167	−0.10	−5.38	0.94	++
3-O-caffeoylquinic acid * (chlorogenic acid)	353.0896	Hydroxycinnamic acids	167	−0.10	−5.39	0.47	++
methyl-3-O-caffeoylquinic acid	367.1049	Hydroxycinnamic acids	154	0.30	−5.56	1.28	+
4-O-caffeoylquinic acid lactone	335.0786	Hydroxycinnamic acids	134	−0.10	−5.67	0.26	+
1,4-O-dicaffeoylquinic acid	515.1208	Hydroxycinnamic acids	211	−0.10	−5.88	0.15	+
ethyl-3-O-caffeoylquinic acid	381.1208	Hydroxycinnamic acids	-	0.70	−5.95	0.74	+
apigenin 7-O-rutinoside	577.1565	Isoflavonoid O-glycosides	225	−0.80	−6.27	1.28	+
methyl-1,5-dicaffeoylquinic acid	529.1361	Hydroxycinnamic acids	200	0.30	−6.28	0.29	+
3-O-caffeoylquinic acid lactone	335.0791	Hydroxycinnamic acid	134	−0.50	−6.37	0.10	+

Asterisks (*) denote compounds confirmed using standards. ^1^ Qualitative classification of BBB permeation potential based on experimental Log(Pe) values: +++ high permeability (Log Pe ≥ −4.5); ++ moderate permeability (−4.5 > Log Pe ≥ −5.5); + low permeability (−5.5 > Log Pe ≥ −6.5). Compounds with Log Pe < −6.5 are considered non-permeant and are not listed.

## Data Availability

Dataset available on request from the authors.

## Supplementary Materials

The following supporting information can be downloaded at: https://www.mdpi.com/article/10.3390/ijms27094059/s1.
