# Supplementary material for: Exploring Neuroprotective Potential of Bioactive Compounds Obtained from Artichoke By-Products by Pressurized Liquid Extraction via Response Surface Methodology
Source: Int J Mol Sci. 2026 Apr 30;27(9):4059. doi: 10.3390/ijms27094059 (PMC13164496; doi:10.3390/ijms27094059)
Supplement: Supplementary file 1 [file ijms-27-04059-s001.zip › ijms-4213188-supplementary.pdf]

Supplementary Tables

Table S1. Analysis of Variance related to the Extraction Yield variable for response surface modeling, presenting linear, quadratic and interaction relations, and coefficient for model prediction.

Source	*Sum of Squares*	*Df*	*Mean Square*	*F-Ratio*	*P-Value*
A: Temperature	28,9521	1	28,9521	54,33	0,0179*
B: %EtOH	25,6267	1	25,6267	48,09	0,0202*
AA	0,0164274	1	0,0164274	0,03	0,8768
AB	0,3844	1	0,3844	0,72	0,4852
BB	3,41195	1	3,41195	6,40	0,1271
Lack-of-fit	1,24025	3	0,413418	0,78	0,6056
Pure error	1,06587	2	0,532933		
Total (corr.)	61,096	10			

Df (degree of freedom)
*Denotes statistical differences ($p<0.05$)

R-squared = 96,2254 percent
R-squared (adjusted for d.f.) = 92,4508 percent
Standard Error of Est. = 0,730023
Mean absolute error = 0,391132
Durbin-Watson statistic = 2,30287 (P=0,4774)
Lag 1 residual autocorrelation = -0,200594

Table S2. Analysis of Variance related to the TPC variable for response surface modeling, presenting linear, quadratic and interaction relations, and coefficient for model prediction.

Source	*Sum of Squares*	*Df*	*Mean Square*	*F-Ratio*	*P-Value*
A: Temperature	16579,5	1	16579,5	95,28	0,0103*
B: %EtOH	155,042	1	155,042	0,89	0,4448
AA	160,219	1	160,219	0,92	0,4385
AB	457,96	1	457,96	2,63	0,2462
BB	885,632	1	885,632	5,09	0,1527
Lack-of-fit	1049,85	3	349,949	2,01	0,3491
Pure error	348,027	2	174,013		
Total (corr.)	19500,0	10			

Df (degree of freedom)
*Denotes statistical differences ($p<0.05$)

R-squared = 92,8314 percent
R-squared (adjusted for d.f.) = 85,6629 percent
Standard Error of Est. = 13,1914
Mean absolute error = 9,61021
Durbin-Watson statistic = 2,75302 (P=0,7663)
Lag 1 residual autocorrelation = -0,459331

Table S3. Analysis of Variance related to the TFC variable for response surface modeling, presenting linear, quadratic and interaction relations, and coefficient for model prediction.

Source	*Sum of Squares*	*Df*	*Mean Square*	*F-Ratio*	*P-Value*
A: Temperature	4,335	1	4,335	7,70	0,1091
B: %EtOH	19,0817	1	19,0817	33,87	0,0283*
AA	0,0294912	1	0,0294912	0,05	0,8403
AB	5,0625	1	5,0625	8,99	0,0956
BB	0,0294912	1	0,0294912	0,05	0,8403
Lack-of-fit	6,32101	3	2,107	3,74	0,2181
Pure error	1,12667	2	0,563333		
Total (corr.)	36,0073	10			

Df (degree of freedom)
*Denotes statistical differences (p<0.05)

R-squared = 79,3162 percent
R-squared (adjusted for d.f.) = 58,6324 percent
Standard Error of Est. = 0,750555
Mean absolute error = 0,677512
Durbin-Watson statistic = 2,32023 (P=0,4890)
Lag 1 residual autocorrelation = -0,22641

Table S4. Analysis of Variance related to the ORAC assay variable for response surface modeling, presenting linear, quadratic and interaction relations, and coefficient for model prediction.

Source	*Sum of Squares*	*Df*	*Mean Square*	*F-Ratio*	*P-Value*
A: Temperature	20,2033	1	20,2033	65,17	0,0150*
B: %EtOH	63,5701	1	63,5701	205,06	0,0048*
AA	7,98158	1	7,98158	25,75	0,0367*
AB	13,8012	1	13,8012	44,52	0,0217*
BB	8,62353	1	8,62353	27,82	0,0341*
Lack-of-fit	6,27818	3	2,09273	6,75	0,1317
Pure error	0,62	2	0,31		
Total (corr.)	117,586	10			

Df (degree of freedom)
*Denotes statistical differences (p<0.05)

R-squared = 94,1335 percent
R-squared (adjusted for d.f.) = 88,267 percent
Standard Error of Est. = 0,556776
Mean absolute error = 0,667727
Durbin-Watson statistic = 1,51478 (P=0,0844)
Lag 1 residual autocorrelation = 0,172509

Table S5. Analysis of Variance related to the DPPH assay variables for response surface modeling, presenting linear, quadratic and interaction relations, and coefficient for model prediction.

Source	*Sum of Squares*	*Df*	*Mean Square*	*F-Ratio*	*P-Value*
A: Temperature*	5449,31	1	5449,31	182,69	0,0054*
B: %EtOH*	4772,57	1	4772,57	160,00	0,0062*
AA*	1331,01	1	1331,01	44,62	0,0217*
AB	0,038025	1	0,038025	0,00	0,9748
BB*	1034,89	1	1034,89	34,70	0,0276*
Lack-of-fit	1474,1	3	491,367	16,47	0,0578
Pure error	59,6562	2	29,8281		
Total (corr.)	14976,6	10			

Df (degree of freedom)
*Denotes statistical differences ($p<0.05$)

R-squared = 89,7589 percent
R-squared (adjusted for d.f.) = 79,5179 percent
Standard Error of Est. = 5,46151
Mean absolute error = 9,43069
Durbin-Watson statistic = 1,53805 (P=0,0908)
Lag 1 residual autocorrelation = 0,121167

Table S6. Complementary PLE experiments comparing ternary solvent mixtures against the optimal binary solvent system: effects on extraction efficiency and bioactivity endpoints. Extractions performed under fixed conditions (10.34 MPa, 180 °C, 20 min, single cycle) using three solvent systems: (1) EtAc/EtOH 90:10 v/v (binary, adopted for RSM); (2) EtAc/EtOH/H_2O 33:33:33 v/v/v (ternary water-containing); (3) EtAc/EtOH/CPME 33:33:33 v/v/v (ternary non-polar). Extraction yield expressed as percentage of dry starting material (%, dry basis) and mass per unit dry extract (mg/g d.e.). Chemical composition measured as Total Phenolic Content (TPC, mg gallic acid equivalents per gram dry extract), Total Flavonoid Content (TFC, mg quercetin equivalents per gram dry extract), and antioxidant capacity via ORAC and DPPH assays (IC_{50} in µg/mL extract). Biological activity assessed via enzyme inhibition assays: AChE, acetylcholinesterase; BuChE, butyrylcholinesterase; LOX, lipoxygenase inhibitory activity (IC_{50} in µg/mL extract). Asterisk (*) refers to standard reference compounds: Trolox (antioxidant control); Galantamine and Quercetin (positive controls for biological activity). Values reported as mean ± standard error of the mean (n = 3).

**Sample**	**Extr. Yield**	**TPC**	**TFC**	**ORAC**	**DPPH**	**AChE**	**BuChE**	**LOX**
	mg/g d.e. (%)	mg GAE/g d.e.	mg QE/g d.e.	IC_{50}, (µg/mL extr.)		IC_{50} (µg/mL extr.)		
**EtAc/EtOH (90:10)**	7.7	165.8 ± 2.9	11.2 ± 0.7	1.1 ± 0.1	123.0 ± 1.5	528.2 ± 13.2	405.7 ± 25.0	279.3 ± 15.1
**EtAc/EtOH/H_2O (33:33:33)**	8.6 ± 0.4	142.4 ± 1.2	9.2 ± 0.5	10.4 ± 0.5	161.0 ± 1.8	596.4 ± 8.1	459.7 ± 15.0	314.6 ±12.5
**EtAc/EtOH/CPME (33:33:33)**	7.9 ± 0.7	137.9 ± 2.9	8.3 ± 0.2	7.6 ± 0.4	134.2 ± 0.6	615.6 ± 16.9	441.4 ± 11.7	302.6 ± 9.8
Trolox*				0.63 ± 0.08	4.12 ± 0.10			
Galantamine*						0.8 ± 0.2	3.6 ± 0.2	
Quercetin*								12.5 ± 0.5

Table S7. Total chlorophyll and total carotenoid content in ABP PLE extracts obtained using three different solvent systems under identical PLE conditions (10.34 MPa, 180 °C, 20 min, single cycle): evidence of solvent-dependent pigment recovery and degradation. Units: chlorophyll content in μg/g dry weight (d.e.); carotenoid content in μg β-carotene equivalents per 100 g d.e.

Sample	Chl a	Chl b	Chl c	Chl total	Total carotenoid content
	(μg/g d.e.)			(μg/g d.e.)	(μg β-carotene eq/100g d.e.)
**EtAc/EtOH (90:10)**	9.8 ± 0.6	2.1 ± 0.1	3.2 ± 0.1	15.1 ± 0.8	1.9 ± 0.1
**EtAc/EtOH/H_2O (33:33:33)**	8.4 ± 0.3	1.6 ± 0.2	2.6 ± 0.2	12.6 ± 0.7	2.1 ± 0.1
**EtAc/EtOH/CPME (33:33:33)**	7.3 ± 0.2	1.7 ± 0.1	2.0 ± 0.1	11.0 ± 0.4	1.5 ± 0.0

Supplementary Figures

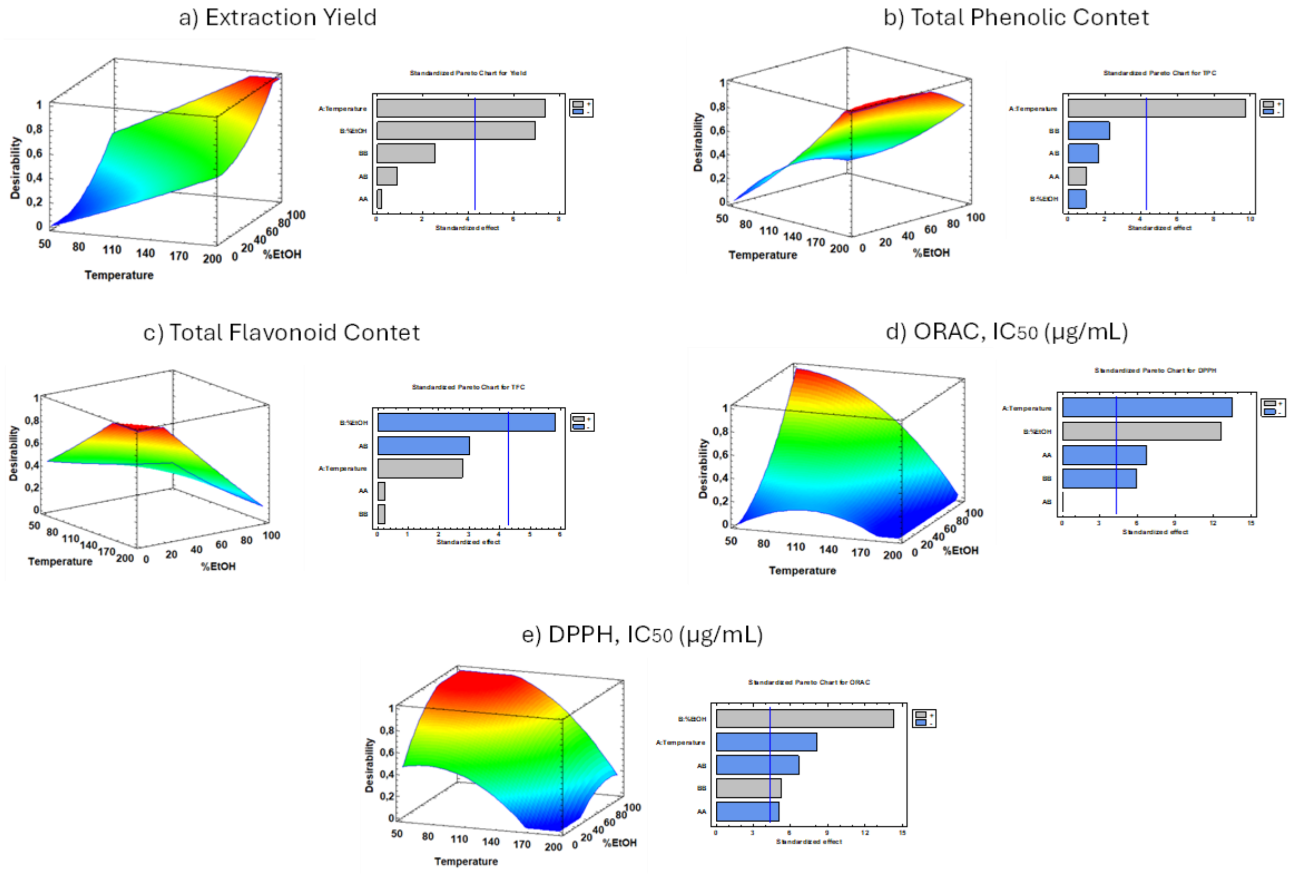

Figure S1. Estimated response surfaces model and their corresponding Standardized Pareto charts for each response variables: a) Extraction yield (%); b) TPC (mg GAE/g d.w.); c) TFC (mg QE/g d.w.); d) IC_{50} ORAC (µg/mL); e) IC_{50} DPPH (µg/mL).

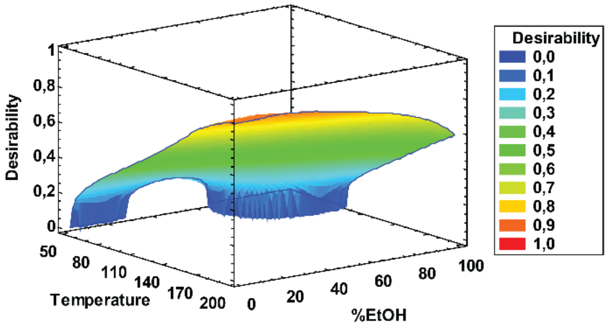

Optimize Desirability
Optimum value = 0,843671

Factor	*Low*	*High*	*Optimum*
Temperature	50,0	180,0	180,0
%EtOH	0,0	100,0	10,4563

Response	*Optimum*
Yield	7,55923
TPC	166,886
TFC	10,3623
ORAC	1,08877
DPPH	119,21

Figure S2. Response surface desirability of optimization of all the response variables.

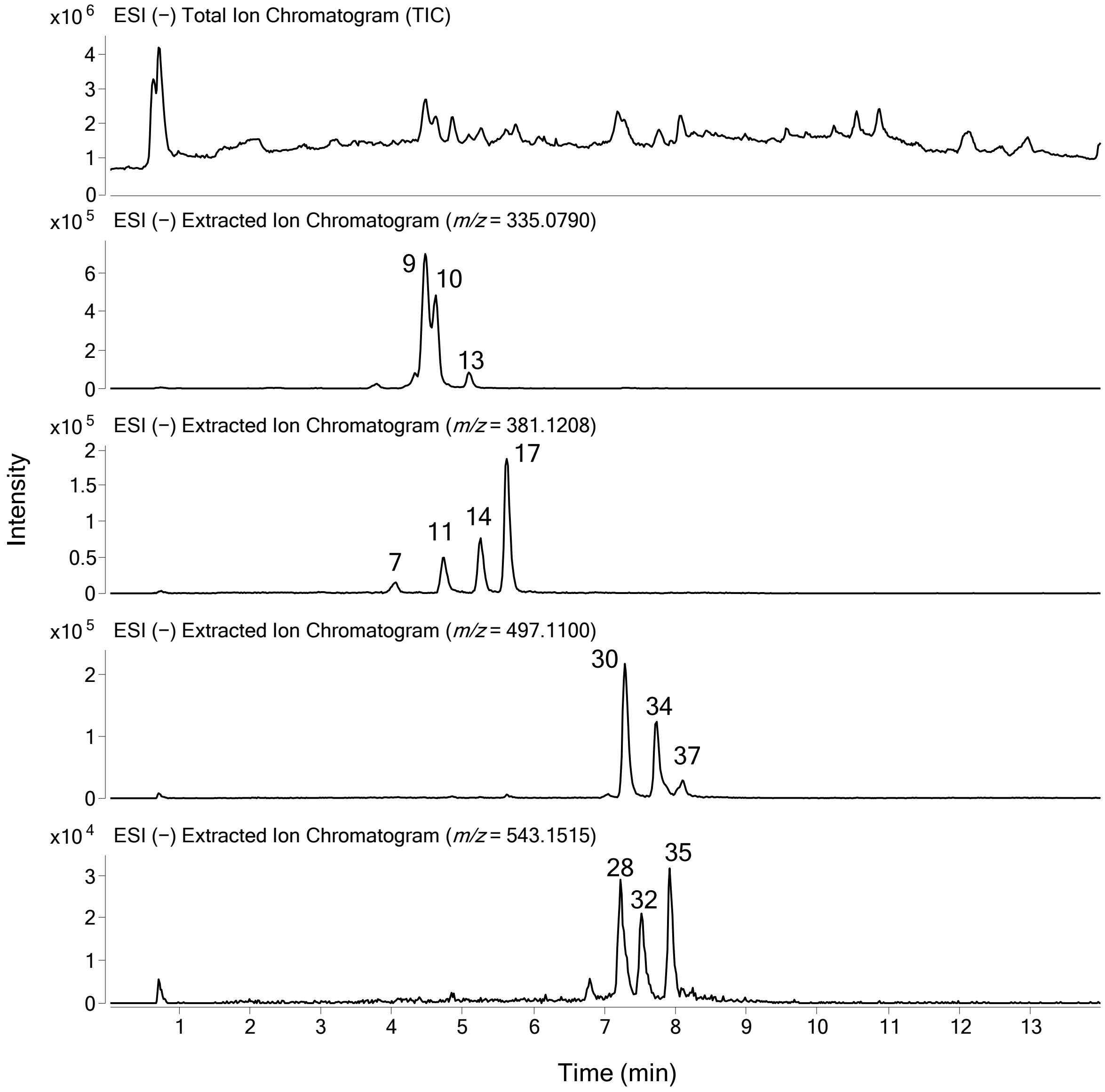

Figure S3. Total Ion Chromatogram (TIC) and Extracted Ion Chromatograms (EIC) of the newly identified compounds in artichoke by-products extracts analyzed by HPLC-C18-Q-TOF MS/MS.

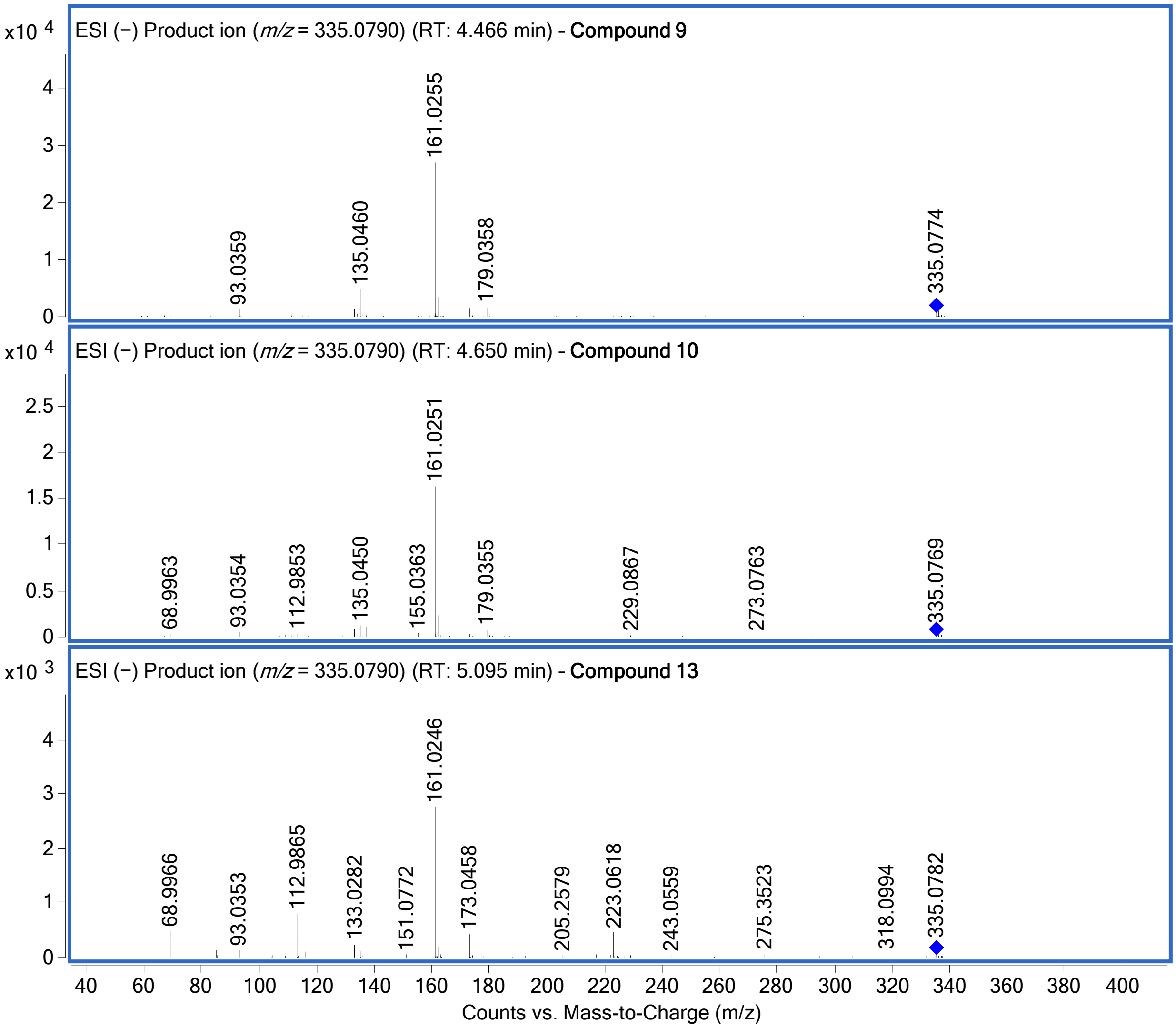

Figure S4. MS/MS spectra of compounds tentatively identified as 3-O-caffeoylquinic acid lactone (**compound 9**), 4-O-caffeoylquinic acid lactone (**compound 10**) and 5-O-caffeoylquinic acid lactone (**compound 13**) in artichoke by-products extracts analyzed by HPLC-C18-Q-TOF MS/MS.

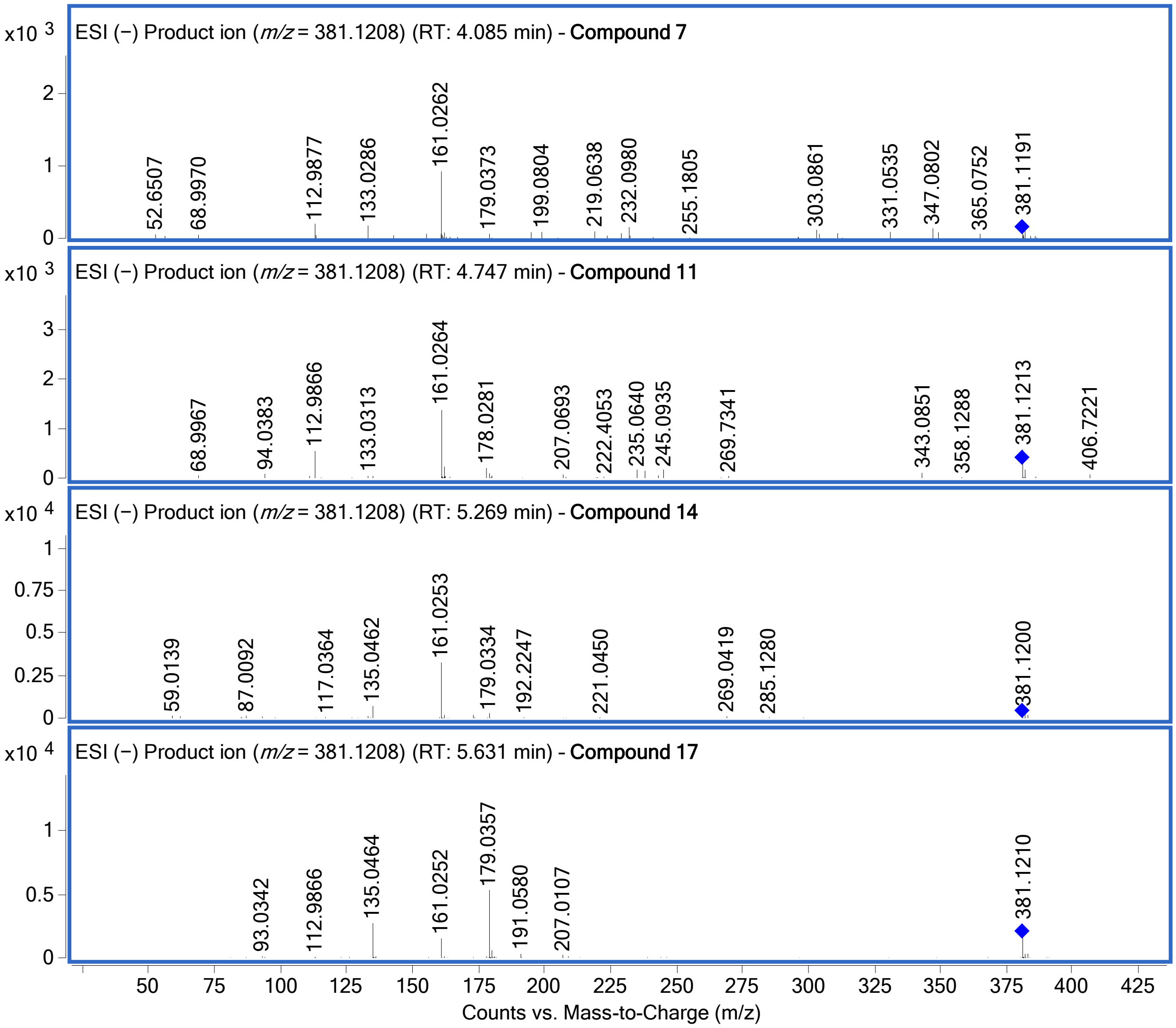

Figure S5. MS/MS spectra of compounds tentatively identified as cis-ethyl-3-O-caffeoylquinic acid (**compound 7**), ethyl-3-O-caffeoylquinic acid (**compound 11**), ethyl-4-O-caffeoylquinic acid (**compound 14**) and ethyl-5-O-caffeoylquinic acid (**compound 17**) in artichoke by-products extracts analyzed by HPLC-C18-Q-TOF MS/MS.

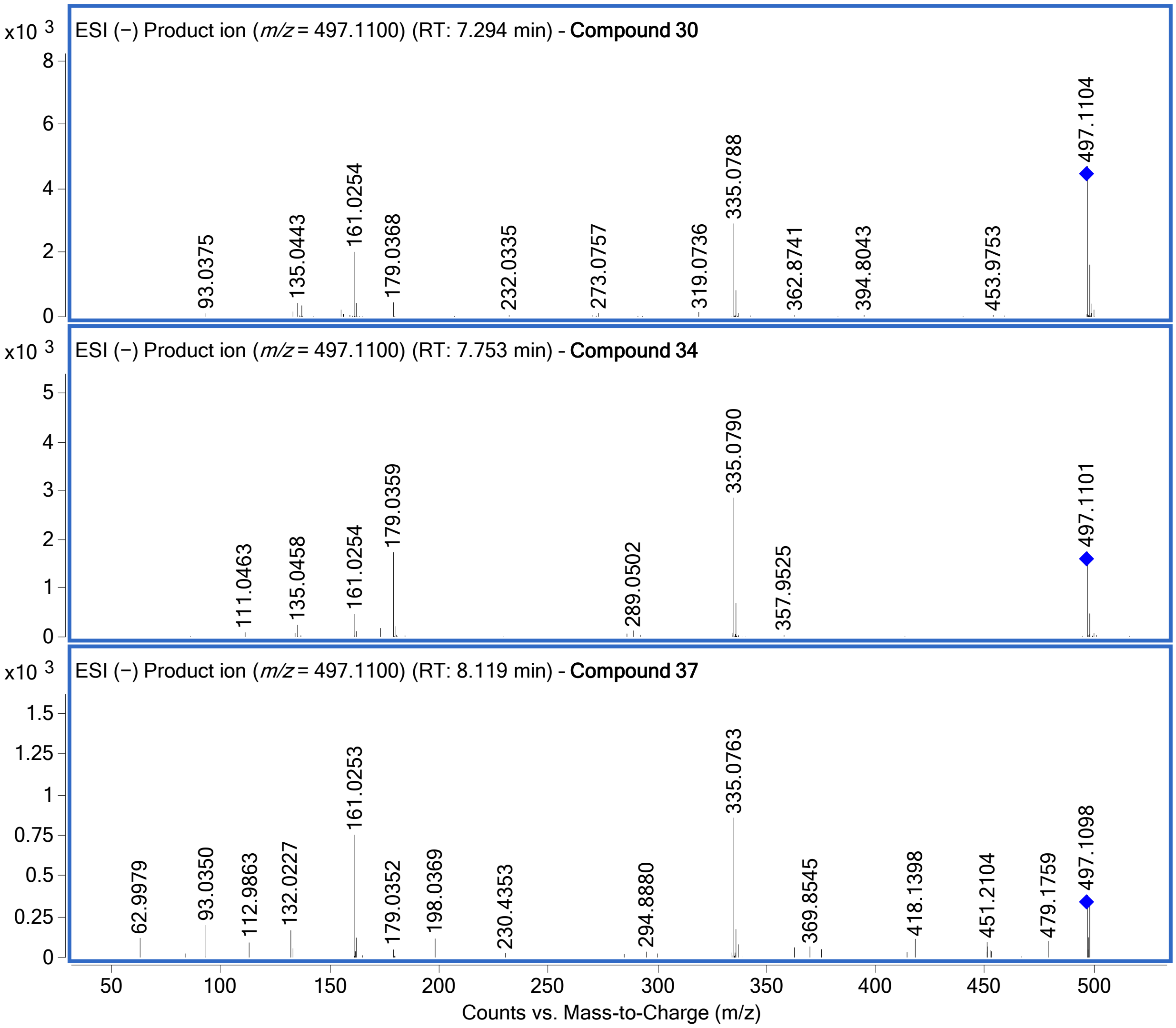

Figure S6. MS/MS spectra of compounds tentatively identified as 3,5-dicaffeoylquinic acid lactone (**compound 30**), 1,5-dicaffeoylquinic acid lactone (**compound 30**) and 4,5-dicaffeoylquinic acid lactone (**compound 37**) in artichoke by-products extracts analyzed by HPLC-C18-Q-TOF MS/MS.

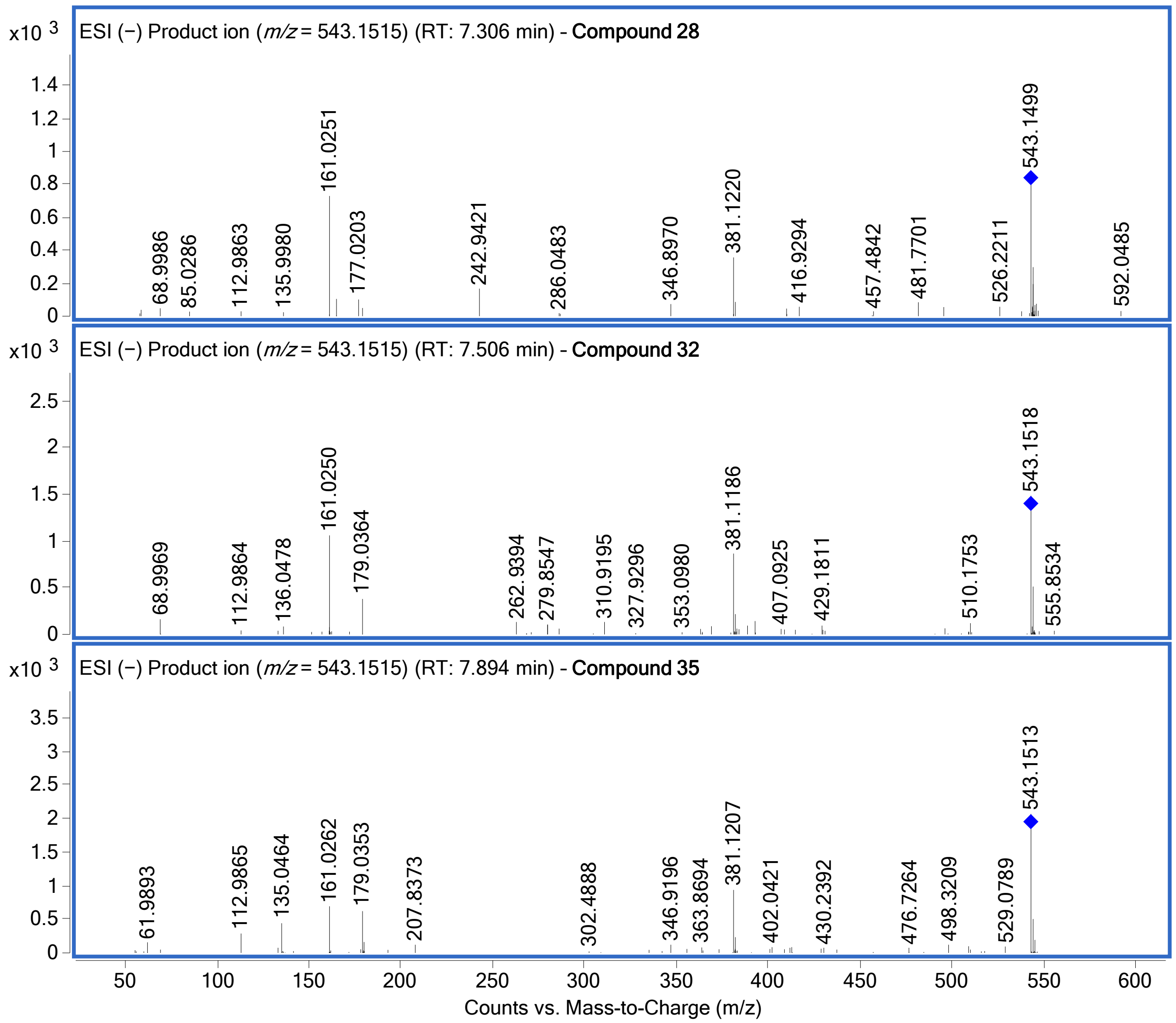

Figure S7. MS/MS spectra of compounds tentatively identified as ethyl-3,5-dicaffeoylquinic acid (**compound 28**), ethyl-1,5-dicaffeoylquinic acid (**compound 32**) and ethyl-4,5-dicaffeoylquinic acid (**compound 37**) in artichoke by-products extracts analyzed by HPLC-C18-Q-TOF MS/MS.
